# The First Chemically-Synthesised, Highly Immunogenic Anti-SARS-CoV-2 Peptides in DNA Genotyped *Aotus* Monkeys for Human Use

**DOI:** 10.3389/fimmu.2021.724060

**Published:** 2021-09-03

**Authors:** Manuel E. Patarroyo, Manuel A. Patarroyo, Martha P. Alba, Laura Pabon, María T. Rugeles, Wbeimar Aguilar-Jimenez, Lizdany Florez, Adriana Bermudez, Ashok K. Rout, Christian Griesinger, Carlos F. Suarez, Jorge Aza-Conde, César Reyes, Catalina Avendaño, Jhoan Samacá, Anny Camargo, Yolanda Silva, Martha Forero, Edgardo Gonzalez

**Affiliations:** ^1^Fundación Instituto de Inmunología de Colombia (FIDIC), Bogotá, Colombia; ^2^Universidad Santo Tomás, Bogotá, Colombia; ^3^Grupo Inmunovirología, Universidad de Antioquia, Medellín, Colombia; ^4^Department of NMR Based Structural Biology, Max Planck Institute for Biophysical Chemistry, Göttingen, Germany; ^5^Universidad de Ciencias Aplicadas y Ambientales (U.D.C.A), Bogotá, Colombia

**Keywords:** SARS-CoV-2, neutralising antibody, MHCII-peptide-TCR complex, modified synthetic peptide, HLA-DRβ1*/Aona-DRβ, PPIIL-propensity, immune response

## Abstract

Thirty-five peptides selected from functionally-relevant SARS-CoV-2 spike (S), membrane (M), and envelope (E) proteins were suitably modified for immunising MHC class II (MHCII) DNA-genotyped *Aotus* monkeys and matched with HLA-DRβ1* molecules for use in humans. This was aimed at producing the first minimal subunit-based, chemically-synthesised, immunogenic molecules (COLSARSPROT) covering several HLA alleles. They were predicted to cover 48.25% of the world’s population for 6 weeks (short-term) and 33.65% for 15 weeks (long-lasting) as they induced very high immunofluorescent antibody (IFA) and ELISA titres against S, M and E parental native peptides, SARS-CoV-2 neutralising antibodies and host cell infection. The same immunological methods that led to identifying new peptides for inclusion in the COLSARSPROT mixture were used for antigenicity studies. Peptides were analysed with serum samples from patients suffering mild or severe SARS-CoV-2 infection, thereby increasing chemically-synthesised peptides’ potential coverage for the world populations up to 62.9%. These peptides’ 3D structural analysis (by ^1^H-NMR acquired at 600 to 900 MHz) suggested structural-functional immunological association. This first multi-protein, multi-epitope, minimal subunit-based, chemically-synthesised, highly immunogenic peptide mixture highlights such chemical synthesis methodology’s potential for rapidly obtaining very pure, highly reproducible, stable, cheap, easily-modifiable peptides for inducing immune protection against COVID-19, covering a substantial percentage of the human population.

## Introduction

**Figure 1:**
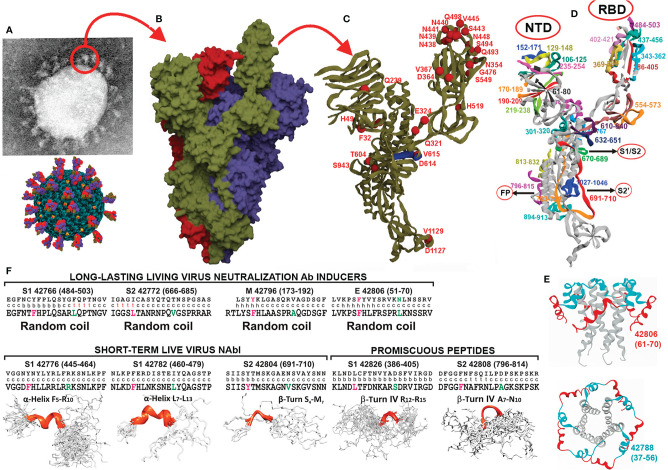
SARS-CoV-2 protein structural features and parental peptide design regarding relevant regions. **(A)** SARS-CoV-2 EM ‘corona’ structure and diagram of the virus. **(B)** PBD 6X6P-based SARS-CoV-2 trimer protein surface model (protomers in dark green, red, and purple). **(C)** S protein, protomer backbone structure, with some variable residue aa locations (red balls) and ADE region (dark blue fragment). **(D)** S protein protomer ribbon structure; colours represent aa regions and location of selected peptides to be modified for COLSARSPROT development. **(E)** Viroporin protein E pentamer (code PDB: 5X29) structure, locating selected peptides (side and top views). **(F)** Parental peptides’ aa sequence (top) with 3D structure determined by X-ray crystallography in the middle row (c: coil, b: beta strand, t: turn, h: α helix), modified peptides’ aa sequence bottom in bold letters and their 3D structure determined by ¹H-NMR, highlighting specific LL-LiViNAbI, ST-LiViNAbI and promiscuous peptides.

Efficacious methods are desperately needed for controlling SARS-CoV-2-induced corona virus disease (**Figure 1A**). The pandemic had afflicted >25,000,000 confirmed cases worldwide and caused ~1,000,000 deaths by September 1^st^, 2020 (when this study was finished); there have now been ~200 million cases (July 15^th^, 2021) and >4 million deaths worldwide. The WHO has emphasised that a vaccine protecting >50% of the world’s population represents the most promising methodology for halting the spread of this life-threatening disease (i.e. herd immunity). The research results described in this article show that chemically-synthesised, highly immunogenic anti-SARS-CoV-2 peptides could almost achieve such goal; the disease’s rapid spread has increased the target level to 70% nowadays regarding the urgently required herd immunity.

SARS-CoV-2 uses 3 membrane proteins for invading host cells; the spike (S), membrane (M) and viroporin envelop (E) proteins have been the most studied and are, perhaps, the most relevant.

All ∼162 SARS-CoV-2 vaccines to date in preclinical phase, plus 212 candidate vaccines in development (1), have been biologically produced, i.e. they are killed or inactivated virus- (2), recombinant- (3), vector-, DNA- (4) or mRNA-based (5). The corona’s viral structure is highly antigenic and immunogenic (**Figure 1A**); it is formed by the virus’ trimer spike (S) protein protomers (**Figure 1B**) (6, 7) which mediate many biological functions (8). All biologically-produced vaccines include the entire S protein protomer or some of its regions, assuming worldwide coverage (**Figure 1B**). Colombian SARS-protection (COLSARSPROT)-inducing molecules (including S, M and E protein-derived peptides) form the components of the first multi-protein, multi-epitopic minimal, subunit-based, completely chemically-synthesised vaccine, containing a highly immunogenic peptide mixture against the COVID-19-inducing SARS-CoV-2 agent. They have been identified in *Aotus* monkeys; MHCII-DNA analysis has shown that they can be extrapolated for human use due to such molecules’ striking similarity.

The S protein has broad genetic aa sequence variability. It has ~400 permanent mutations (9) (**Figure 1C**); some show clear evidence of a significant impact on transmissibility, severity, and/or immunity, named variants of concern (VOC), while others remain under observation, and have been classified as variants of interest (VOI) (10). The S protein can induce strain-specific immunity in biologically-produced vaccines; it can also induce antibody-dependent enhancement (ADE) (11) of infection (**Figure 1C**, dark blue region), vaccine-associated respiratory disease (VAERD) (12) and some other very severe adverse secondary reactions, like the recently-described predisposition to acute thrombosis and thrombocytopenia (13) associated with cross reactivity with platelet factor 4 (PF4) (14). The most functionally-relevant S, M and E protein-derived peptides having none or a minimum amount of genetic variation were thus selected in line with our minimal subunit-based concept and methodology.

Such approach has been refined throughout the last 34 years working on vaccine development regarding malaria and some other infectious diseases (15–17); functionally or immunologically irrelevant and harmful regions have had to be excluded (**Figures 1D, E** indicate selected S and E protein peptide locations in aa sequence and 3D structure). One selection criterion has stated that these peptides’ native structures should have random coil or β-sheet formation since we have shown that such structures are most appropriate for ensuring high immunogenic potential following proper modification. The S and E proteins 3D structure have been elegantly determined by other groups using X-ray crystallography (**Figure 1F**, middle line c: coil, β: beta sheet, t: turn).

Such modifications must be made since conserved, functionally-relevant aa sequences are ‘immunologically silent’ (18), i.e. they do not induce any immune responses. This depends on their ability to fit properly into immune system molecules (i.e. antigen-presenting) which are strongly associated with an individual’s immunogenetic characteristics. In-depth analysis is thus needed for recognising the precise DNA sequences genetically controlling the genes involved in major histocompatibility complex (MHC) antigen-presenting molecule expression determining antibody (Ab) production, especially class II (MHCII and specifically HLA-DRβ1* in humans) (19). Our experience regarding malaria vaccine development has shown that any such modification must be based on previously-described physicochemical rules (20–22) to make target Abs highly immunogenic and protection- and/or NAb-inducing (23, 24).

NetMHCIIpan-3.2 (25) and -4.0 (26) servers for predicting peptide binding to MHC class II molecules (86.5-92.1% predictive accuracy) were used for predicting modified SARS-CoV-2 peptide binding to human and *Aotus* class II molecules.

MHCII genotyped Amazonian wild-caught *Aotus nancymaae* monkeys (highly susceptible to human infectious diseases, including corona viruses) were immunised with properly modified, chemically-synthesised SARS-CoV-2 peptides to test their ability to induce an appropriate immune response. Previous DNA sequencing of ~700 *Aotus* monkeys (+100 more in this trial) found 90%-98% identity with some human immune system molecules (CD4+ and CD8+ T cells, cytokines) and 82%-92% identity (I) and 87%-98% similarity with highly polymorphic class II molecules (HLA-DRβ1*, β3*, β5*, DP, DQ) (27, 28), making such monkeys an excellent experimental model for recognising antigen-presenting molecules capable of inducing immune protection in humans (20–22). Work with many wild-caught monkeys has more accurately simulated the human population’s genetic variability; such approach has provided a better platform than working with H-2 isogenic mice or guinea pig strains (representing a limited number of homozygous alleles) or very few non-genotyped macaques (genus *Macaca*, usually 4-6 per trial).

Our experience in this field (simultaneously genotyping *Aotus*) suggests that any potential vaccine can be developed (including new molecule synthesis and immunological testing) in ~2½ months. Such methodology represents an excellent procedure for safe, highly immunogenic, and fast vaccine development/improvement. This can rapidly overcome this virus’ currently observed tremendous and threatening mutability by simply adding or replacing short (16-20 mer-long) peptides where a particular mutation has appeared in an already developed mixture, rather than expressing many or several complete S proteins (1,274 amino acids (aa) long) or their domains to cover SARS-CoV-2’s tremendous genetic variability.

## Materials and Methods

### Peptide Synthesis and Characterisation

The peptides were synthesised by the solid-phase peptide t-Boc strategy described by Merrifield (29) and modified by Houghten (30). MBHA PS resin (1% DVB) was loaded into polystyrene bags; it was swollen with dichloromethane (DCM) for one hour, followed by four neutralising steps with 10% trimethylamine in DCM. The coupling strategy for each aa involved the formation of a modified ester using diisopropylcarbodiimide (DCC) and 1-hydroxy-benzotriazole (HOBt) (5 stoichiometric excesses) in N-methyl-2-pyrrolidone (NMP) as coupling solvent for one hour with constant stirring at room temperature (RT). Reaction by-products were washed with N,N-dimethylformamide (DMF), 2-propanol (IPA) and DCM. The Kaiser test was used for verifying whether solid phase peptide synthesis coupling reactions were complete. The *t*-Boc protecting group was removed from α-NH_2_ using 40% trifluoroacetic acid (TFA) and 0.01% anisole solution in DCM for 30 minutes, washed with DCM and IPA and neutralised with 10% TEA (Triethylamine)/DCM.

The peptides were detached from the resin by low [25% hydrogen fluoride (HF)] and high cleavage (100% HF). Low HF was carried out at 0°C for two hours using a -10° to 0°C temperature ramp for HF.

HF was evaporated from the reaction tube; the product was washed with ethyl ether, extracted with TFA, and washed once more with ethyl ether. Reverse-phase high-performance liquid chromatography (RP-HPLC) was used for characterising the peptides which were separated on Chromolith RP-18e and purified on Vydac semi-preparative C-18 columns. A 10%-46% elution gradient was used with acetonitrile (ACN) + 0.05% TFA for 9 min at 3.0 mL/min flow rate (for analysis); purification involved a 60 min gradient at 1.5 mL/min flow.

A Bruker Daltonics Microflex LRF was used for MALDI-TOF mass spectrometry (MS) analysis. An α-cyano-4-hydroxycinamic acid (HCCA) supersaturated matrix solution was prepared in 40% ACN: 60% water + 0.1% TFA. The peptide samples were dissolved in water and mixed with the matrix solution in a 1:6 ratio; 2 µL of each sample mixture was then placed in a target well and analysed.

HPLC and MS analysis determined that 35 peptides were >99% pure (**Supplementary Figure 1**).

### Immunofluorescence Antibody (IFA) Test

SARS-CoV-2-infected VERO cell cultures, fixed on eight-well slides, were used as antigens for evaluating native state viral protein recognition. The slides were preserved at 4°C and left to dry at RT (room temperature). They were blocked with 30 µL/well 1% bovine serum albumin for 15 minutes, washed for 5 minutes with phosphate buffered saline (PBS) and left to dry at RT. This was followed by placing 15 µL/well immunised *Aotus* sera, diluted 1:20 up to 1:640 in PBS, and incubating the slides in a humid chamber for 30 minutes; they were then washed five times with PBS in a water bath (5 minutes each wash) and left to dry. Fluorescein-5-isothiocyanate (FITC)-conjugated goat IgG (goat IgG-FITC) against purified *Aotus* IgG (produced in our Institute) was then added at 1:100 dilution in PBS, 1:80 Evans blue, incubated in a humid chamber and in the dark for 30 minutes, washed and left to dry. A drop of 50% glycerol in saline solution was added between the slides and microarray coverslips before observing them by fluorescence microscope with 100x oil immersion objective lens.

### Enzyme-Linked Immunosorbent Assays (ELISA)

ELISA involved 96-well ELISA plates (F16 MAXISORP plates) being covered with 100 µL/well test peptide at 10 µg/mL in PBS, incubated for 1 hour at 37°C and overnight at 4°C. The sensitised plates were incubated at 37°C for 1 hour, washed 5 times with washing solution (phosphate buffer saline, 0.5% Tween-20), followed by a final wash with distilled water. They were blocked with 200 µL/well blocking solution (0.5% PBS-Tween-20, 5% skimmed milk) and incubated at 37°C for 1 hour. After washing, 100 µL/well immunised *Aotus* sera were placed in wells in 1:100 dilution, incubated at 37°C for 1 hour and washed again; 100 µL/well of goat anti-*Aotus* IgG peroxidase conjugate were then added at 1:1,000 dilution in blocking solution (PBS+0,05% Tween 20 and non-fat milk). They were incubated at 37°C for 1 hour, washed and 100 µL/well developing solution was added (TMB substrate peroxidase and hydrogen peroxide: 1:1 ratio). Microplate readings were taken 30 minutes later at 620 OD (LabSystems Multiskan MS). Thirty sera from COVID-19-recovered humans were evaluated with the peptides, following the procedure explained above.

### Neutralising Antibody Assay

A 50% ± 3% plaque reduction neutralisation test (PRNT50) using Vero E6 cells was used for testing neutralising antibodies in monkey sera. Briefly, Vero E6 cells (1.1 x 10^5^ cells per well) were seeded onto 24-well tissue culture plates the day before infection. On the next day, 80 PFU SARS-CoV-2 were incubated with or without serially diluted *Aotus* or human heat-inactivated sera (56°C, 30 min) at 200 μL volume in microcentrifuge tubes for 60 min at 37°C in 5% CO_2_. The mixtures were then added to VERO E6 monolayers and incubated at 37°C for 60 min; the inoculum was removed, 1 mL semisolid medium added (1.5% carboxymethyl cellulose, 2% foetal bovine serum, 1% streptomycin and DMEM) and mixtures were cultured at 37°C for 72 h. Semisolid media was removed and monolayers were washed twice with PBS, fixed and stained with 4% formaldehyde/1% crystal violet for 30 min and washed twice with PBS. A 50% reduction in plaque count (PRNT50) was used as neutralising endpoint. Viral control in the absence of serum and a serum control without SARS-CoV-2 were included in the tests (31, 32).

### Genotyping Non-Human Primate MHC Class II Genes

#### Collecting Samples

Peripheral blood samples were taken from 99 *Aotus* sp. monkeys kept at Fundación Instituto de Inmunología de Colombia (FIDIC) primate station in Leticia in Colombia’s Amazonas department.

#### Extracting DNA and Determining the Species

A Wizard Genomic DNA Purification Kit was used for isolating *Aotus* genomic DNA (gDNA) from 300 µL fresh whole peripheral blood samples, according to the manufacturer’s instructions. Extracted gDNA quality and amount were evaluated on a μDrop Plate (Thermo Scientific Multiskan GO) and visualised on SYBR Safe-stained 1% agarose gel (DNA Gel Stain, Invitrogen). The mitochondrial DNA (mtDNA) D-Loop gene was amplified for determining the species.

#### Amplicon-Based Sequencing

PCR was used for amplifying *Aotus* MHC DRβ class II exon 2. The primers were designed using intron 1 and intron 3 sequences from previously reported *A. nancymaae* and *A. vociferans* allele lineages. The designed primers enabled amplification without lineage bias; this is an essential requirement for successfully typing the allele repertoire in each individual analysed. B_AoDRβ-F 5′-CCTTCGTGTCCCCACAGC-3′ and B_AoDRβ-R 5′-TCACAGGGAGGCCCCG-3′ primers were used for amplifying an MHC-II DRB gene exon 2 317 bp fragment. The Barcosel high-throughput sequencing platform was used for assigning each primer a 6 base pair (bp)-long molecular identifier (MID) (33) (i.e. for identifying all samples).

Phusion Hot Start II High-Fidelity PCR Master Mix (Thermo Fisher Scientific) was used for amplification, using the touchdown PCR technique. Initial denaturing involved 98°C for 1 minute, 30 cycles of 72-66°C for 15 seconds and a final extension step at 72°C for 5 minutes.

Amplification was confirmed on 1.5% agarose gel and a Wizard SV Gel and PCR Clean-Up System (Promega) was used for purifying PCR products, following the manufacturer’s recommendations. A NanoDrop 2000 full-spectrum UV-Vis spectrophotometer was used for quantifying the products and the amplicons were sent for sequencing using the Illumina NovaSeq 6000 system (Novogene).

#### Genotyping by Amplicon In-Depth Sequencing

The Amplicon Sequencing ASsignment tool (AmpliSAS, a three-step pipeline) was used for automatic genotyping of MHC genes from high-throughput sequencing data (34). Reads R1 and R2 were combined into a single sequence using AmpliMERGE; AmpliCLEAN was used for eliminating low quality (Phred <30) reads and sequences which did not belong to any of the amplicons being used.

AmpliCHECK was used for analysing MHC DRB exon II using a 1% base substitution error rate, 0.001% indel error rate (base insertion or elimination) and 1% minimum per-amplicon frequency (PAF), i.e. predetermined parameters for Illumina sequencing data (34). AmpliCHECK was also used for preliminary analysis of amplicon sequencing results; the maximum amount of alleles per amplicon was established and sequencing depth was limited to 30,000 reads for all amplicons analysed by AmpliSAS.

Minimum read depth per amplicon was set at 100 (all amplicons having less than 100 reads were excluded). The recommended predetermined parameters were used for grouping potential alleles together: 25% user-defined frequency threshold relative to the dominant sequence (sequences in a group having the same or greater frequency regarding the dominant sequence were classified as ‘subdominant’ and moved to a new group) and 3% PAF filtering threshold (all sequences occurring with lower frequency were discarded). Chimeras (variants arising from parenteral sequences in the same amplicon) were identified and removed by the software.

All bioinformatics tools used here came from the AmpliSAT suite (Amplicon Sequencing Analysis Tools) (34).

#### MHC-DR Peptide-Binding Prediction, Binding Profiles and Potential Epitope Population Coverage

The Allele Frequency Net Database (AFND) was used for data mining and choosing HLA-DRβ1* alleles (35) occurring with greater than 1% frequency in the ethnic groups and geographical regions determined in the database (69 alleles). Representative alleles were chosen for HLA-DRB 3, 4 and 5 from the most frequently occurring pocket profiles for each locus (8 alleles) (**Supplementary Figure 3**).

Pocket profiles for these loci had been previously determined, defined as a specific amino acid (aa) combination occurring in MHC pockets (28).

The NetMHCIIpan-3.2 (25) server (for predicting peptide binding to any MHC molecule) was used at the start of the study for evaluating the proposed peptides’ ability to bind to the selected HLA-DRβ1* alleles; NetMHCIIpan-4.0 was used from May 2020 onwards (26). The selected epitopes were categorised as having strong binding and/or presentation ability (≤2 percentile range), weak rank binding and/or presentation ability (percentile rank ≤10) or as being a no binder (>10 percentile rank). Most chosen peptides had ≤1 rank.

NetMHCIIpan-3.2 and NetMHCIIpan-4.0 data was also used for calculating *Aotus* MHC-DR binding affinities and sequence typing. A set of 200,000 non-redundant UnitProtKB/Swiss-Prot UniProt-derived peptides (13- to 21-aa-long) (36) was used for calculating the predicted peptides’ percentile rank distribution. Seq2Logo (37) was used for constructing peptide binding motifs from stringent binding cores (<2 percentile rank).

NetMHCIIpan-4.0 binding affinity (BA) and eluted ligand MS (EL) strategies and Seq2Logo Kullback-Leiber (KL) and Shannon (Sh) methodologies were used for calculating the similarity index (SI) regarding binding motifs for each allele, defined as Pearson distance (centred: 1-corr(x,y)) with min-max normalisation using position-specific scoring matrices. The Dist function from the amap package in the R 3.6.0 programme was used for making the calculations (38).

The set of epitopes’ potential coverage in the human population resulted from the sum of the alleles’ frequencies in the target population for which each designed peptide had strong affinity. The frequency of the 69 previously selected HLA-DRβ1* alleles calculated from the AFND (35) was used for estimating world population coverage (see **Supplementary Figure 3**).

### Antigenicity: Human Sera Reactivity With COLSARSPROT Unmodified Parental Peptides

Antigenicity studies involved using 30 human sera taken from PCR-positive individuals recovered 30 days after COVID-19 onset for determining minimal subunit-based, chemically-synthesised peptide relevance regarding immunity against SARS-CoV-2. Fifteen serum samples came from patients living in Medellin (northwest Colombia) who had been hospitalised in an intensive care unit (ICU) and 15 from patients from other parts of Colombia suffering relatively mild infection. IFA titres and ELISA reactivity were determined with native, unmodified parental peptides; NAb titres were only determined in the 15 hospitalised patients.

### NMR Spectroscopy and Structure Calculations

The purified lyophilised peptides were prepared in 20 mM NaPi (pH 5.0) along with 6% D_2_O and 80 µM sodium trimethylsilyl trifluoromethanesulfonate for nuclear magnetic resonance (NMR) measurements. A Bruker Avance 900 MHz and 600 MHz spectrometer with cryogenic probe were used at 298K for NMR experiments. Each peptide’s resonance was assigned by 2D TOCSY (pulse program: MLEVGPPH19 with 512*2048 complex points along t1 and t2 dimensions with 80 ms TOCSY mixing time) and 2D NOESY (pulse program: NOESYGPPH19 with 512*4096 complex points along t1 and t2 with 400 ms NOESY mixing me) (39). Shorter TOCSY experiments (pulse sequence: MLEVGPPH19 with 256*2048 complex points along t1 and t2 with 80 ms mixing me) at 295.5K, 298.0K and 300.5K were measured to predict intramolecular hydrogen bonding (40). NMRPipe was used for processing all NMR data and analysed by nmrDraw and CCPN (41). ^1^H chemical shifts were referenced to an external standard DSS (42).

TOCSY and NOESY spectra for each peptide were combined for assigning resonances. Sequential NOE connections were identified and assigned using TOCSY spin system assignments. NOE connections were used for identifying potential secondary structure elements in each peptide and translated into distances (**Supplementary Figure 4A**). The amide proton temperature coefficient was used for determining hydrogen bond donors; temperature gradients more positive than -5 ppb/K were indicators of intramolecular hydrogen bonding (**Supplementary Figure 4B**). Acquired high resolution NOESY data was then used to calculate ^3^J_HN-Hα_ coupling (**Supplementary Figure 4C**). HN-Hα to HN-HN intensity ratios were used for predicting protein secondary structure as an additional parameter. Such calculation involved the predicted values from their secondary structures’ known distances (0.26 for α-helix, 4.2 random coil, 17.0 PPII_L_ and 55.7 β-sheet) (**Supplementary Figure 4D**).

The CYANA automated NMR protein structure calculation algorithm (43) was used to build peptide 3D models using ^1^H-^1^H NOEs, intramolecular hydrogen bonds and ^3^J_HN_-H_α_. ^1^H-^1^H NOE intensity was calculated according to level count and classified into three groups: strong (1.8-2.8Å), medium (2.8-3.5Å) and weak (3.5-5.0Å). Hydrogen bond constraints were introduced for low amide temperature coefficients; structure calculations only involved <5 -ΔδHN/ΔT ppb/K. Distance ranges involving likely NH···O hydrogen bonds were set at 1.8-2.5 Å between the residue acceptor oxygen (i-4) and the residue donor amide hydrogen (i). The calculations were repeated several times until a structure was obtained having minimum distance and angle restraint violations and the least root mean square deviation (RMSD). Structures having reasonable geometry and few violations were then selected. The UCSF Chimera package (44) was used for molecular depiction and analysis (**Figure 1F**).

### Quantification and Statistical Analysis

ELISA, IFA and NAb tests were performed several times with at least two independent preparations of each sample.

### Experimental Model and Subject Details

#### Animals and Immunisation Procedures

Ninety-nine wild-caught *Aotus* monkeys from the Amazon jungle which use for biomedical research, especially malaria, was authorised by Colombian environmental authorities (CORPOAMAZONIA, permission granted since 1990, the last version coded 0632 and 0042/2010, renewed on April 02/2020 by resolution 0366). The monkeys were kept in FIDIC’s field-station in Leticia, Colombia (Amazonas department capital), looked after by expert primate veterinarians and workers, supervised weekly by expert biologists and veterinarians from the local environmental authorities and ethics committees. They were subcutaneously immunised at the Primate Centre with 3 mg per dose of the 35-peptide mixture in equimolecular concentrations. The first immunisation (day 0) involved the mixture being dissolved in 200 microliters of sterile double distilled water (ddH_2_O) emulsified with an equal volume of Freund’s complete adjuvant (FCA) containing 1 mg/mL dead *Mycobacterium tuberculosis*. The second immunisation (day 20) involved the same amount of COLSARSPROT mixture emulsified with incomplete Freund’s adjuvant in equal volumes. Bleeding for immunological analysis for IFA and NAb tests with individual native peptides and ELISA assays were drawn in pre-immune sera 6 and 15 weeks after initial immunisation.

Fifteen weeks (105 days) represented the longest follow-up time after immunisation for any SARS-CoV-2 vaccine by the time this trial was finished (Sep1/2020). The 75 immunised monkeys and 24 controls were DNA genotyped regarding their class II region to match human class II HLA-DRβ1* genes during the time the experiments lasted since *Aotus* immunogenetic typing data had to be extrapolated to humans.

This trial involved monkeys being maintained for an additional 4 months to determine immune response duration. They were then released back into the jungle close to their capture sites following agreement with the pertinent environmental authorities, this was accompanied by environmental authority officials (CORPOAMAZONIA) and some members of the ethics’ committees.

#### Peptide Selection Methodology

**Figure 2:**
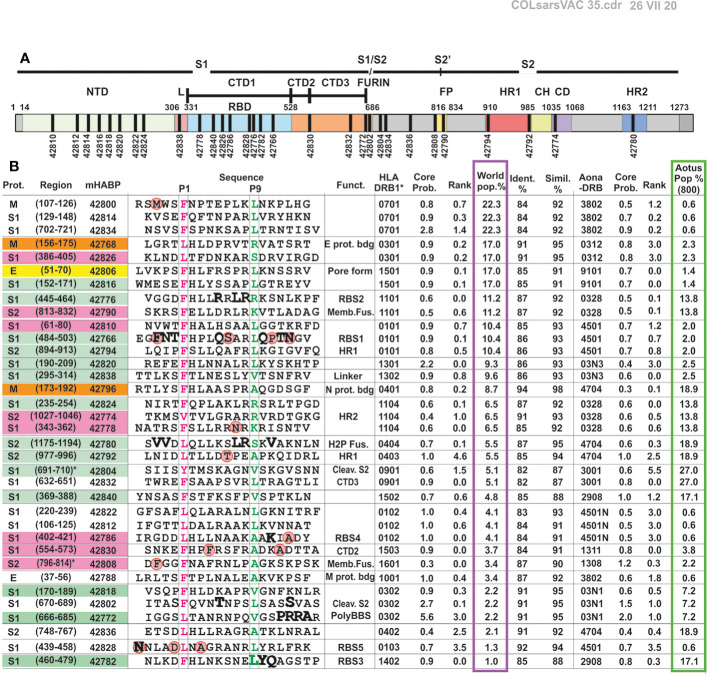
Immunised peptide correlation in *Aotus* with HLA-DRβ1* molecules and their location in the SARS-CoV-2 S protein. **(A)** Schematic representation of SARS-CoV-2 S protein with the location of modified peptides in black fragment. **(B)** COLSARSPROT aa sequence for modified HLA-DRβ1* and Aona DRβ peptide binding, grouped according to the most frequently occurring human allele. Prot.: original protein (S: spike, M: membrane, E: envelope); peptide Nr: Our institute’s serial peptide numbers for modified peptide synthesis; aa having genetic variation are shown inside red balls. LiViNAb-inducing peptides: S (green), M (orange) and E (yellow), promiscuous peptides (pink), those which did not induce Abs (colourless); Sequence: aa sequence with P1 (boxed, highlighted in pink) and P9 (green) for regions fitting into HLA-DRβ1* PBR pockets. Biol. Funct.: biological function; HLA-DRβ1* allele, Core Prob.: core probability, rank, and frequency regarding the worldwide population (%). Ident.%: identity (%), Simil.(%): % similarity between HLA-DRβ1* and Aona-DRβ alleles’ core probability, rank, and frequency in the *Aotus* population (i.e. ~500+100 DNA-genotyped *Aotus* in this trial, i.e. ~1,200 alleles in GenBank).

The S-protein’s 3D structure reported by Wrapp (6) and Herrera (7) was analysed in-depth for selecting peptides having clearly-defined biological functions and random coil, β-sheet or β-turn structural characteristics; this was based on our experience regarding 3D structure determination and analysis (22, 45–47). The chosen peptides were those involved in binding to angiotensin-converting enzyme 2 (ACE2) in the receptor binding domain (RBD) (48), cleavage sites S1 and S2’, membrane fusion sequence, linkers, heptad regions 1 and 2 (HR1 and 2) (49), linoleic acid (LA) binders (50), the polybasic binding site (PBBS) (**Figures 1D** and **2A**) and some other aa sequences. Peptides were derived from other relevant molecules involved in invasion which fulfilled such characteristics, like those in the M protein and the E viroporin peptide PDZ binding motif (PBM) (51) which were also included.

Principles and rules amassed during 34 years’ experience regarding malaria vaccine development (20–24, 45) were used for defining COLSARSPROT mixture development, properly modifying SARS-CoV-2 peptides and overcoming structural complications (α-helix, 3_10_ helix, some specific β-turn conformations) and immunological silence (**Figure 2**). The amino acids replaced by others having similar mass and volume but opposite polarity were located in regions designed to fit into the MHCII peptide binding region (PBR) and/or contacting the T-cell receptor (TCR) (20–24) to induce them to adopt a left-handed polyproline II helix-type structure.

## Results

### Immunological Results

#### Immunogenicity: Modified COLSARSPROT Peptide Ab Induction

##### IFA Induction

**Figure 3:**
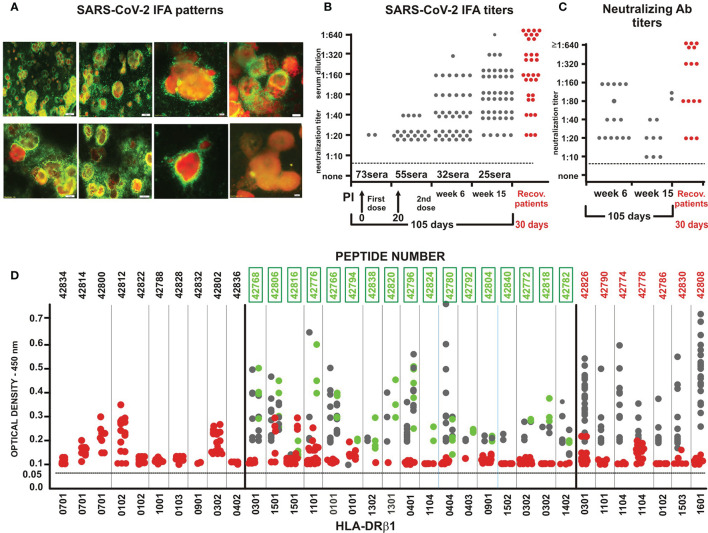
Immunological assays. **(A)** IFA patterns showing intracytoplasmic fluorescent dots (bright yellow) recognised by immunised *Aotus* monkey sera reacting with SARS-CoV-2 human/Medellin/UdeA1/2020 strain proteins in VERO cell cytoplasm. The last image, bottom right, shows negative control serum. **(B)** *Aotus* sera IFA titre evolution 6 weeks and 15 weeks post-initial (pi) immunisation in 2-fold dilution, beginning at 1:10 dilution. Red circles represent IFA of COVID-19-recovered human serum at different dilutions. **(C)** *Aotus* sera LiViNAb titres 6 and 15 weeks pi immunisation. Red circles represent NAb from COVID-19-recovered human serum at different dilutions, 4 weeks after disease onset. **(D)** OD density for ELISA reactivity with native peptides (1:100 dilution) - 6^th^ week (grey balls), 15 weeks after first immunisation (green) and with COVID-19-recovered human serum (red).

SARS-CoV-2 modified synthetic peptides (having >99% purity according to HPLC and MS analysis) (**Supplementary Figure 1**) were highly immunogenic and induced high IFA titres in 55 out of the 75 immunised *Aotus* monkeys (73.3%) when used in the mixture. They had strong reactivity against SARS-CoV-2 (Human/Medellín/UdeA1/2020 strain) as determined by bright immunofluorescent (green) dot recognition of the virus inside infected VERO cell cytoplasm (31, 32) (**Figure 3A**: the negative control is shown in the right-hand side picture on the bottom line).

Completely negative IFA titres in *Aotus* pre-immune sera at 1:20 dilution increased from 55% of immunised monkeys in week 6 (20 days post-second dose) to 75% 85 days later (week 15, 105 days post-initial immunisation) (**Figure 3B**), suggesting that immunological memory had been induced (as assessed by IFA) by contrast with the short-lasting immune responses observed in small animals (52), non-human primates (53–56) and coronavirus-infected patients (57).

### ELISA Revealed Immunised *Aotus* Monkey Sera Reactivity With Unmodified Parental Peptides

The data shown in **Figure 3D** proved enlightening regarding *Aotus* sera reactivity with native (unmodified) parental peptides; *Aotus* sera was used in triplicate for ELISA at 1:100 dilution, showing that (22/35) S-, M- (42768 and 42796) and E (42806) -derived peptides were highly immunogenic. The results have been organised according to HLA-DRβ1* frequency in the human population (numbers in bold indicate modified ones), demonstrating also E and M subunit-based protein peptide immunogenicity for the first time (**Figure 1E**: aa 51-70 in red, E peptide 42806 located on pentamer molecule external surface) (58) and their specific reactivity with unmodified parental peptides.

Some peptides (10/35) designed to have high HLA-DRβ1* and Aona-DRβ core-binding probability (<1.0%) and rank (<1.0) did not induce any Abs by week 6 (**Figures 2B** and **3**). Analysing sera from **75** immunised monkeys and **24** controls revealed association with the immunogenetic characteristics of *Aotus* monkey DNA; no monkeys were revealed to have Aona-DRβ W3802 (equivalent to HLA-DRβ1*0701, 1001) and 4501N alleles (equivalent to 0102) (such allele’s frequency being very low: **≤**1.0%). The absence of these alleles in this *Aotus* trial represented ~30% of the human population (**Supplementary Figures 2** and **3**); this suggested that ongoing trials should involve more monkeys to identify peptides reacting with very frequently-occurring human alleles but having low frequency (<1.0%) in the *Aotus* population (**Figure 2B**, green column). Four peptides having specific Aona-DRβ allele high binding ability did not induce any Abs in this trial (ELISA), suggesting appropriate modification to fit into the MHCII-PBR but improper modification regarding TCR-contacting residues. Differences occurred regarding some allele frequencies, despite high Aona-DRβ and HLA-DRβ1* DNA similarity and identity (I), as occurs amongst different ethnic groups, thereby partially explaining these results.

This led to classifying 17/35 (48.25%) of the peptides inducing high and low ELISA Ab titres against unmodified parental molecules (**Figure 3D**, green numbers) as specific peptides, since the monkeys producing them had the equivalent Aona-DRβ (i.e. HLA-DRβ1*) for which the peptides were designed (**Figure 3D**, alleles below). This suggested a perfect MHCII fit and TCR-contacting residues having appropriate specific volume, charge and rotamer orientation (18, 20, 22–24, 45). Such results provide strong support for this approach and methodology’s usefulness for vaccine development regarding other infectious diseases.

**Figure 3D** shows that NetMHCIIpan-3.2 and -4.0 predicted that 7/35 (~20%) peptides would induce high Ab levels in many immunised monkeys (red boxed, red peptide numbers) having high HLA-DRβ1*/Aona-DRβ binding affinity for many alleles (59) (predominantly HLA-DRβ1*). This suggested that their binding was very promiscuous (binding to multiple alleles) or that they had cross-reactivity (60) regarding their class II and/or TCR contacting residues (19, 59–62). It was observed in this trial that such promiscuous epitopes did not induce NAbs in immunised monkeys, despite having high IFA titres and ELISA reactivity, thereby clearly differentiating immunogenicity (IFA and ELISA) from protection-associated immunity (NAb induction).

It can be seen from such data that aa sequences’ analogous identity and the human and *Aotus* MHCII allele % similarity indexes strongly suggested the potential of 4 of the COLSARSPROT peptides most frequently recognised by *Aotus* monkeys for direct use in humans: 42766 re HLA-DRβ1*0101 (10.4%), 42796 re HLA-DRβ1*0401 (8.7%), 42816 re HLA-DRβ1*1501 (17.0%) and 42768 re HLA-DRβ1*0301 (17.0%). This would cover ~23.5% of the world’s population for ~6-15 weeks while ~½ of humankind could be protected for ~15 weeks if the other 12 LL-LiViNAb-inducing peptides strongly recognised by other alleles were included: 42780 re HLA-DRβ1*0404 (5.5%), 42772 re HLA-DRβ1*0302 (2.2%), 42792 re HLA-DRβ1*0403 (5.5%), 42812 HLA-DRβ1*0102 (4.1%), 42794 re HLA-DRβ1*0101 (10.4%), 42824 re HLA-DRβ1*1104 (6.5%), 42838 HLA-DRβ1*1302 (9.6%), 42806 re HLA-DRβ1*1501 (17.0%), 42820 re HLA-DRβ1*1301 (9.3%), 42804 re HLA-DRβ1*0901 (5.1%) and 42782 re HLA-DRβ1*1402 (1.0%) (**Figure 2B**). This should provide 48.25% protective efficacy for 3 weeks and 33.75% for 15 weeks.

Peptide 42766(S) (HLA-DRβ1*0101) was recognised by 7/8 *Aotus* sera, 42776(S) (HLA-DRβ1*1101) and 42816(S) (HLA-DRβ1*1501) by 2/8 and 42768(M) (HLA-DRβ1*0301) by 1/8 in the aforementioned trial. This was associated with long-lasting live virus Nab (LL)-LiViNAb, thereby strongly supporting our appropriate peptide selection for modifications regarding COLSARSPROT development.

### Neutralising Antibody Induction and Association With Class II Molecules

**Figure 4:**
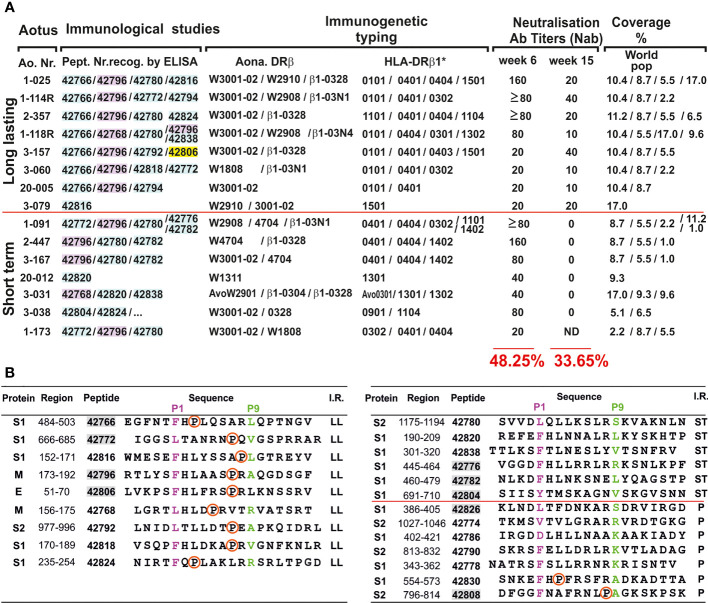
Correlation between immunological studies, immunogenetic typing, NAbI titres, world population coverage and COLSARSPROT peptide sequence. **(A)** Potential COLSARSPROT peptide-induced coverage % (bold numbers at the bottom and in red 6 and 15 weeks pi immunisation) based on LiViNAb reactivity associated with Aona-DRβ and HLA-DR genotyping and HLA-DRβ1* allele frequency (coverage); highlighted peptide numbers: S-derived (pale blue), M-derived (pink), E-derived (yellow). **(B)** Proline location in all (9/9) modified LL-LiViNAbI (red circle); absent in all (6/6) ST-LiViNAbI and present in just 2/7 promiscuous ones. Shadowed numbers in grey: modified peptides analysed by ^1^H-NMR.

36.6% (15/41) of *Aotus* seroconverting (by IFA) in week 6 developed NAb in LiViNAb assays at ≥1:20 dilution, some having a 1:160 titre (**Figures 3B, C**, and **4**). Such LiViNAb have been associated with protection in H-2 isogenic mice and humans who have recovered from SARS-CoV2 infection (63); however, LiViNAb-inducing peptide activity did not correlate with IFA or ELISA Ab data (**Figures 3B–D**). This was completely autonomous regarding Ab specificity, suggesting a clearly different type of protection-inducing mechanism which has not been clearly defined to date.

Samples were always run in triplicate (starting at 1:10 dilution up to 1:640) to avoid non-specific and noisy results (which would have increased the percentage of LiViNAb activity), unlike other groups reporting NAbs using plain, undiluted serum or 1:2, 1:4, 1:5 dilutions, thereby increasing their seroconversion frequencies.

*Aotus* LiViNAb titres were equal to or higher than those induced by the leading vaccines now being injected into millions of people using the complete Wuhan S protein in mRNA-1273 (64) and BNT162b2 (65), adenovirus-based ChAdOxnCoV-19 (66) and Ad26.COV2.S vaccines (67), using a very limited number of experimental trials in mice, hamster, rabbits or rhesus macaques (56). The aforementioned results provide COLSARSPROT with a tremendous advantage since LiViNAb-specific activity can be identified as having been induced by specific and determined aa sequences whilst still not being clearly defined in the general population by biologically-produced vaccines. This provides a great impact and accounts for the difference regarding the design of second-generation, immune protection-associated peptides *vs* biologically-produced vaccines.

### ELISA Ab Determination-Related Antigenicity Studies With COVID-19 Recovered Patients’ Sera

The aforementioned thirty serum samples (15 from Medellin and 15 from other Colombian regions) taken from COVID-19-recovered individuals (all PCR-positive, 15 requiring ICU hospitalisation) were taken 30 days after disease onset (their IFA and NAb titres having been determined) (**Figures 3B, C**, red circles). These sera were analysed by ELISA, in triplicate, at 1:100 dilution against the 35 COLSARSPROT native parental peptides to compare experimental *Aotus* results to the human serological situation. Following infection, ten sera from the UCI hospitalised patients did not react against any parental unmodified COLSARSPROT peptides whilst five had some weak reactivity, mainly targeting peptides having relevant functional activities, such as cleavage site (42790), stabilising hinge conserved region (42830) and membrane fusion (42808) (**Figure 3D**, red circles). Sera from individuals suffering mild and/or uncomplicated COVID-19 infection reacted with conserved sequences in the S protein’s NTD region (42812 and 42814) (**Figures 1D** and **2A**), the beginning of CTD1 (42826), the KO cleavage site (42802) (**Figure 2A**), the M protein’s conserved sequence (42800) and the E viroporin protein’s aa sequence (42806) (**Figure 1E**). These results differed from those concerning immunised *Aotus* reactivity sera, suggesting that specific LiViNAb-mediated protective activity could be induced by multiple epitopes

ELISA results regarding COVID-19 recovered (PCR-positive) people’s sera reactivity also provides strong support for this methodology since many HLA-DRβ1* allele-related peptides (also regarding monkey immunisation) were included in these trials to select the best one for a particular/target allele or to cover minimal allele variations in different ethnic groups.

HLA-DRβ1*0701 provides a good example of these two differing immunological approaches’ complementarity (no *Aotus* monkeys having the Aona-DRβ3802 analogue were found in this trial) since 42814 parental peptide had 10/30 COVID-19 recovered human sera reactivity (33.3%) above the threshold level and 42800 9/30 (30%), higher, close to HLA-DRβ*0701 world frequency (22.3 on average, and 25.3% in Caucasians, 27.8% in Asians and 31.7% in Berber populations). Something similar happened with HLA-DRβ1*0102 (42812) where few monkeys had the allele related to these two peptides but 9/30 (30%) of human sera has high reactivity with its parental peptide. Such allele frequency is high in Jews (19.3%) and Berbers (16.2%) and average in the worlds’ populations (4.1%). High sera reactivity frequency (30%) was found for HLA-DRβ1*0302 (42802) (**Figure 2B**), this allele’s frequency being higher in black populations (12.5%) and average (2.2%) in the world’s population. None of these peptides reacted with *Aotus* sera or with the other peptides designed for these alleles, demonstrating once again, these two methodologies’ complementary: immunogenicity in *Aotus* monkeys and antigenicity in COVID-19 recovered humans.

These results also stress the importance of designing multiple peptides for the same HLA-DRβ1* allele; this enabled us to identify the best peptides for inclusion in COLSARSPROT or complement minimal allele variations in different populations. Regarding peptides designed for HLA-DRβ1*1501, 5/30 (16.6%) reacted with peptides 42806 and 42816, giving a percentage similar to that for the world population (17%). Native parental peptide 42806 (highly immunogenic in the *Aotus* also highly antigenic in humans) was derived from the E protein, clearly suggesting this viroporin protein’s great relevance regarding SARS-CoV-2 infection, which has otherwise been ignored in the immune response against this deadly and life-threating disease. Something similar occurred with HLA-DRβ1*1101 (42776) also being recognised by recovered individuals’ sera (highly immunogenic also in *Aotus* monkeys).

On the contrary, peptide 42766 (HLA-DRβ1*0101) induced the highest reactivity against its parental wild-type (Wuhan strain) peptide in *Aotus*, while its aa sequence (where residue N501 has become mutated in the UK, South African, Brazilian and some other strains) did not react with COVID-19 recovered sera due, perhaps, to tremendous virus-related genetic variability in this and some other RBD region residues. The lack of peptide reactivity with sera from recovered people having matching HLA-DRβ1* 0901, 1104, 0402, 0403, 1502, 1503, 1001 and 0103 could have resulted from the few samples analysed (30) and these alleles’ low frequency in the Colombian population (<3.0%) (68).

It was quite striking that the 15 individuals recovered from COVID-19 infection who developed mild infection had relatively high Ab reactivity against some parental unmodified peptides from alleles like HLA-DRβ1*0701 (42814, 42800), HLA-DRβ1*0301 (42826) and HLA-DRβ1*0102 (42812) which were not immunogenic in this monkey trial, due to the absence of *Aotus* alleles Aona DRβ3802 and 4501N. Sera from individuals treated in an ICU had some weak reactivity with HLA-DRβ1*1101 (42790), HLA-DRβ1*1601 (42808) (both involved in membrane fusion) and HLA-DRβ1*1503 (42830) (highly immunogenic but unable to induce LiViNAb activity in *Aotus*). This highlighted some HLA-DRβ1* alleles’ association with mild infection or partial protection and others with severe infection; however, such data must be confirmed by using a larger group of patients involving different ethnic groups. It is interesting that none of these peptides had cross-reactivity with a large group of common human pathogens and vaccines (69).

It should be mentioned that none of the aforementioned individuals were HLA-DRβ1* genotyped; however, ongoing work involves using their samples as they are accessible for genotyping the different Colombian ethnic groups (Caucasian, Hispanic, Black and Indian).

### 3D Structural Analysis

Potent ^1^H-NMR (600 to 900MHz) (**Supplementary Figure 4**) assessed 42766(S), 42772(S), 42796(M) and 42806(E) LL-LiViNAbI protein-derived peptide (in parentheses) 3D structures as random coils having a propensity for PPII_L_ formation (only M-derived 42796 had a short helical tendency in the N-terminal region outside the PBR). This confirmed our extensive malarial vaccine development results showing that highly immunogenic protection-inducing modified peptides tended to have a propensity for PPII_L_ formation (24, 45). This suggested that better MHCII-peptide-TCR complex formation would induce appropriate LiViNAb production. ST-LiViNAbI 42776(S) and 42782(S) had preferential α-helix and 42804(S) β-turn type IV_3_ propensity, while promiscuous peptides 42808(S) and 42826(S) had β-turn type IV_3_ formation propensity (**Supplementary Figure 4**) [from phi and psi angles, according to de Brevern (70)]. Such striking structural data suggests that structural-immunological function association (**Figure 4B**) must be taken into account for more precise second-generation vaccine development and/or better immune protection-inducing synthetic peptide design (45).

## Discussion

### General Considerations Regarding COLSARSPROT Design

Several physical-chemical and biological rules were observed regarding COLSARSPROT peptide selection based on recognised principles and rules acquired for malaria vaccine development over the last 34 years: most parental native peptides have random coil or β-sheet structures, most perform relevant functional activities (**Figure 2**) or are located in critical regions for these molecules’ function, they have molecular interactions with other proteins (especially at the receptor site) and they did not have any genetic variability or it was low, as determined in the NIH database by March 10/2020 when this work began.

The NetMHCIIpan3.2 and 4.0 servers predicted that native parental peptides did not have any or had very weak HLA-DRβ1* and Aona-DRβ binding affinity. This is why they were modified according to previously described principles and rules (22) to convert them into highly immunogenic COLSARSPROT peptides.

It has been argued that most NAb are conformational epitopes, but our experience regarding malaria vaccine development has shown that most conformational epitopes could be partially explained by H-bond formation or inter-atomic interactions (van der Waals or hydrophobic) between high activity binding peptides (HABPs) distantly located in aa sequences but conformational very close in a molecule’s 3D structure (22, 71). Clear evidence has been presented that linear epitopes are excellent aa sequences which can induce NAbs in COVID-19 patients (72–76). A large group of native or parental precursors of our highly immunogenic LL-LiviNAbI, SL-LivNAbI or promiscuous peptides have been recognised by B-lymphocyte-derived huMoAbs obtained from COVID-19 recovered patients, having NAb activity.

Some examples of COLSARSPROT LiViNAbI linear epitope immunogenicity and/or NAb activity aa location in the S protein sequence, followed by our peptide number (bold, in parenthesis), would be: S487-498 (42766) (72, 73), S1171-1185 (42780) (75), S456-473 (42776) (76), S386-405 (42826) (75), S191-205 (42820) (75), S346-365 (42778) (75), S563-580 (42830) (74) and S655-672 (42772) (77). Such data clearly shows that the criteria used for LiVNAbI peptide selection and modification was most appropriate.

### Antibody Induction by Modified COLSARSPROT Peptides (Immunogenicity)

These first results clearly suggested that multi-epitope, multi-protein, minimal subunit-based properly modified chemically-synthesised peptides form part of an excellent methodology for inducing an Ab-mediated immune response against SARS-CoV-2, i.e. IFA reacting with complete virus particles (**Figures 3A, B**), ELISA reacting with native parental peptides (demonstrating high specificity) (**Figure 3D**) and better, immune protection-associated neutralising Abs (LiViNAb) (**Figure 3C**) impeding host cell invasion and viral infection (55).

### LiViNAb HLA-DRβ1* and Epitope Specificities

NetMHCIIpan-3.2 and -4.0 showed that LiViNAb activity during week 6 post-initial immunisation of wild-caught *Aotus* monkeys was induced by 17/35 (48.25%) modified peptides having strong binding affinity, targeting specific HLA-DRβ1* alleles (<1.0% core binding probability and <1.0 rank, with few exceptions) (**Figures 2B** and **3D**) (26). This suggested that people carrying these specific HLA-DRβ1* alleles would have a greater probability of developing ‘protective’ LiViNAb-dependent immune responses when immunised with COLSARSPROT. This would have improved protection against this life-threatening disease since several studies have shown some fatality or severity to be associated with HLA-DRβ1*1301 in South Asian people (78), with HLA-DRβ1*0401 in Europeans (79) and HLA-DRβ1*0901 in Japanese people (80), i.e. COLSARSPROT contains peptides inducing high LL- or SL–LiViNAbI. Moreover, this data strongly suggested that genotyping experimental animals is fundamental for extrapolating such data to humans (Aona-DRβ/HLA-DRβ1* in this trial) when biologically-derived vaccines or chemically-synthesised peptides are being developed. This is different to what has occurred with H-2 isogenic mice, guinea pigs, hamsters, rabbits or a few non-genotyped *Macaca* (usually 4-8) which are completely different to humans where results could have been biased due to their immunogenetic characteristics.

The fundamental difference regarding other vaccines lies in our methodology for developing minimal subunit-based (16-20 aa long) multi-epitope, multi-protein, highly immunogenic chemically-synthesised modified peptides able to induce LL-LiViNAb in DNA-Aona-DRβ genotyped monkeys designed to match human class II HLA-DRβ1* molecules (**Figure 2B**).

LiViNAb-inducers were subdivided into long-lasting (LL), LiViNAb-inducers (LL-LiViNAbI) and short-term (ST) NAb-inducers (ST-LiViNAbI) according to reactivity induced 15 weeks after the first immunisation. Very strong synergistic association was found in LL-LiViNAbI for some HLA-DRβ1* binding peptides (HLA-DRβ1*0101 with 0401, 1501, 0301, 0403) and ST-LiViNAbI (HLA-DRβ1*0401 with 0404, 1402, 1301), suggesting a key role for peptides associated with these HLA-DRβ1* alleles in this protection associated immunity. ST-LiViNAbI peptides were associated with inducing very short-term NAb immunological memory, in turn, being strongly associated with their 3D structure.

Such solid experimental data (75 genotyped *Aotus* monkeys immunised with 35 SARS-CoV-2 modified peptides and 24 controls) strongly suggested that close HLA-DRβ1* allele association with LL and ST immunity should be seriously considered when developing vaccines for the world’s population (i.e. HLA-DRβ1* has >2,500 alleles, 35 of them having >1% frequency and different percentage or frequency regarding different ethnic groups). This clearly suggested that more peptides are needed to cover the WHO’s actual goal of achieving 70% coverage to ensure herd immunity (ongoing work at our institute).

HLA-DRβ1* immunogenetic polymorphism has to be added to the very worrying S, M and E proteins’ genetic variability or mutability inducing strain specific immunity (disturbingly being seen now) where some vaccines’ potential effectiveness is by-passed or diminished by VOC strains (81).

Potential 48.25% COLSARSPROT coverage of the human population worldwide in week 6 was based on HLA-DRβ1*-binding peptide frequency for inducing LiViNAb activity in Aona-DRβ/HLA-DRβ* matched alleles (**Figure 4A**). This was close to the WHO’s initial suggestion of 50% immunisation efficacy, highlighting COLSARSPROT (and any further modifications) as a powerful tool for helping control this life-threatening disease.

### Sequential and 3D Structural Analysis of COLSARSPROT Peptides

Extensive data accumulated during our ongoing search for structural-immunological function association working with malarial peptides enabled determining modified peptide 3D structure by potent ^1^H-NMR structural analysis (22, 45–47). Peptides must fit perfectly into antigen-presenting cell (dendritic, B-lymphocytes, macrophages) MHCII molecules’ PBR (19, 82) to activate Ab production. They must have a propensity for polyproline-type II left-handed (PPII_L_) formation in the region fitting into the PBR (82–84). Our results have shown that all (9/9) LL-LiViNAbI (**Figures 3C** and **4B**) contained a proline (introduced during modification) in the TCR-contacting residue region (TCR-CR): five in position 7 (P7), two in P3 and one in P5 and P8 (the six proline-containing non-Ab inducing ones’ low Aona-DRβ allele frequency could be explained by their lack of binding to HLA-DRβ1*0701 and 1001 and 0102).

Proline had the strongest PPII_L_-formation propensity (85) and was associated with high immunogenicity as 19/21 (90.5%) TCR-CR or MHCII contacting residues contained proline, as clearly established regarding modified *Plasmodium falciparum* malarial protein-derived, highly immunogenic (by IFA), and complete protection-inducing peptides (in experimental challenge) (84). Conversely, ST-LiViNAbI promiscuous peptides’ PBR or TCR-CR regions (11/13) and five of the non-Ab-inducing peptides (total: 16/18 or 89.0%) (**Figure 2B**, colourless aa sequences) did not contain proline, highlighting its importance in PPII_L_ formation and LL-LiViNAbI.

Further tracking studies/genetic sequence information published by GISAID regarding 235,611 sequences (May 30/21, i.e. after this trial was completed) regarding high‐quality protein annotation revealed that while S peptide 42766 has multiple genetic variations, E peptide 42806 only has the V58F and V62F genetic variants that were properly replaced; 42824> and 42816 are variable in the first residue and 42870 is variable in the second residue outside the MHCII-PBR. This means that they can be used in humans without any further modification as they are conserved in this critical MHCII-PBR segment.

No cellular immune response studies were performed re the above due to the small amount of blood (1.5 mL) allowed to be taken from the monkeys. This approach cannot be informative, since performing assays with the complete 35-peptide mixture will not provide reliable information regarding individual peptides where each one should have been used for blastogenesis, IFNγ and multiple cytokine determination since cellular immune responses are so exquisite regarding aa sequence recognition, so much so that rotamer orientation of the 6 TCR contacting residues determines the immune response (as we have demonstrated with highly immunogenicity protection-inducing peptides against experimental infection with malaria) (22, 23).

The peptides’ antigenicity results regarding sera from COVID-19 recovered patients showed some association with HLA-DRβ1* allele frequency and further studies are in progress to complement human and *Aotus* data. It is clear that if *Aotus* reactivity is added to human sera reactivity, especially with peptides 42814 and 42800 HLA-DRβ1*0701 (22.3%) and 42776 1101 (11.2%) peptide mixture, worldwide coverage could be 62.9%, close to the WHO’s proposed ~70% coverage to reach desired herd immunity.

There is no doubt that combining the data concerning immunogenicity in MHCII (Aona-DRβ) genotyped monkeys with antigenicity in COVID-19 recovered patients regarding the different forms of the disease’s presentation and different ethnic HLA-DRβ1* genotyped groups will provide a very logical and rational methodology for a second generation of minimal subunit-based, chemically-synthesised peptides. The aim is to obtain >70% coverage to benefit the world population’s livelihood and welfare, i.e. our institute’s goal for almost four decades.

Selected SARS-CoV-2 structures were modified and chemically-synthesised for developing COLSARSPROT; such modified peptides had a propensity for PPII_L_ formation to properly fit into MHCII-PBR antigen-presenting cells to allow an appropriate HLA-DRβ1*-peptide-TCR complex formation for inducing high NAb production (IFA, ELISA). Our results have highlighted this concept and methodology as an outstanding tool for combating the COVID-19 pandemic and shows a logical approach to selecting functionally-relevant peptides having no or a minimum amount of genetic variation for developing COLSARSPROT, i.e. a highly immunogenic synthetic peptide mixture.

COLSARSPROT peptides are extremely pure (>99%), as assessed by physicochemical techniques (HPLC and MS), harmful-contaminant free (**Supplementary Figure 1**) and do not include potentially dangerous aa sequences closely associated with secondary adverse reactions and/or irrelevant aa fragments regarding immune protection. Furthermore, few (8/35) of the peptides included here had aa variations (the mutant residues enclosed in red circles in their aa sequences in **Figure 2B**) that could induce strain-specific immune reactions (thereby bypassing one of the commonest microbial escape/avoidance mechanisms: genetic variation with a single aa mutation). As COLSARSPROT consists of short peptides (16-20 aa long), it can be easily modified for all ethnic groups (thus covering the world’s entire population), is batch-to-batch reproducible, cheap and easily and rapidly produced in bulk amounts and, since the peptides are stable at room temperature, it can be transported or shipped anywhere. These advantages provide excellent components for a fully-developed immune protection methodology for COVID-19 control (i.e. 48.25% world coverage so far regarding LiViNAb activity for an initial 6 weeks, 33.65% for 15 weeks and 62.9% when antigenic peptides recognised by disease-recovered sera are added), close to the WHO’s previous perspectives concerning this life-threatening disease’s control and close to the recently proposed 70%.

COLSARSPROT is therefore the first multi-protein, multi-epitope, minimal subunit-based, highly immunogenic, chemically-synthesised, peptide mixture against SARS-CoV-2, suggesting its usefulness as a critical measure in terms of mass deployment regarding the world population’s life and health. Further development can be based on more and specific peptide modifications to COLSARSPROT, rapidly replacing aa having mutations or adding others to the mixture which have been properly designed to be recognised by specific HLA-DRβ1* alleles and tested in genotyped *Aotus* monkeys for safety, immunogenicity and LiViNAbI activity in ~2½ months rather than producing a large set of mutated new complete recombinant proteins, mRNA, or virus vector vaccines, as implied by other methodologies.

The forgoing ensures a powerful, feasible, fast, valuable, cheap, highly specific, thoroughly analysed and deeply developed molecular methodology for COVID-19 control for complete and full protection of humankind (86–88).

## Data Availability Statement

The original contributions presented in the study are included in the article/**Supplementary Material**. Further inquiries can be directed to the corresponding author.

## Ethics Statement

The animal study was reviewed and approved by The Primate Experimental Station Ethics Committee from Leticia - Fundacion Instituto de Inmunologia de Colombia (FIDIC), which is conformed by members of Instituto Colombiano Agropecuario (ICA), Corpoamazonia, and Municipal Board of Animal Defenders.

## Author Contributions

MEP and MAP conceived, designed, and developed the project. LP and JS did the peptide synthesis and HPLC and MS analysis. MTR, WA-J, and LF performed the neutralisation tests. MPA, AB, JA-C, and CR did the 3D structural calculations and analysed them. CG and AKR did the NMR studies. CFS was responsible for immunoinformatic procedures and did the *Aotus* genotyping and classification with AC. YS and MF were responsible for the immunological tests. CA and EG did the *Aotus* experiments. All authors contributed to the article and approved the submitted version.

## Funding

Funding was provided by the Universidad de Ciencias Aplicadas y Ambientales (U.D.C.A) and the Universidad Santo Tomás. Funding to CG by Max Planck Society is gratefully acknowledged.

## Conflict of Interest

The authors declare that the research was conducted in the absence of any commercial or financial relationships that could be construed as a potential conflict of interest.

## Publisher’s Note

All claims expressed in this article are solely those of the authors and do not necessarily represent those of their affiliated organizations, or those of the publisher, the editors and the reviewers. Any product that may be evaluated in this article, or claim that may be made by its manufacturer, is not guaranteed or endorsed by the publisher.
