# Supplementary material for: The First Chemically-Synthesised, Highly Immunogenic Anti-SARS-CoV-2 Peptides in DNA Genotyped Aotus Monkeys for Human Use
Source: Front Immunol. 2021 Sep 3;12:724060. doi: 10.3389/fimmu.2021.724060 (PMC8446425; doi:10.3389/fimmu.2021.724060)
Supplement: Supplementary file 1 [file DataSheet_1.pdf]

Supplementary Material

Supplementary Figure 1

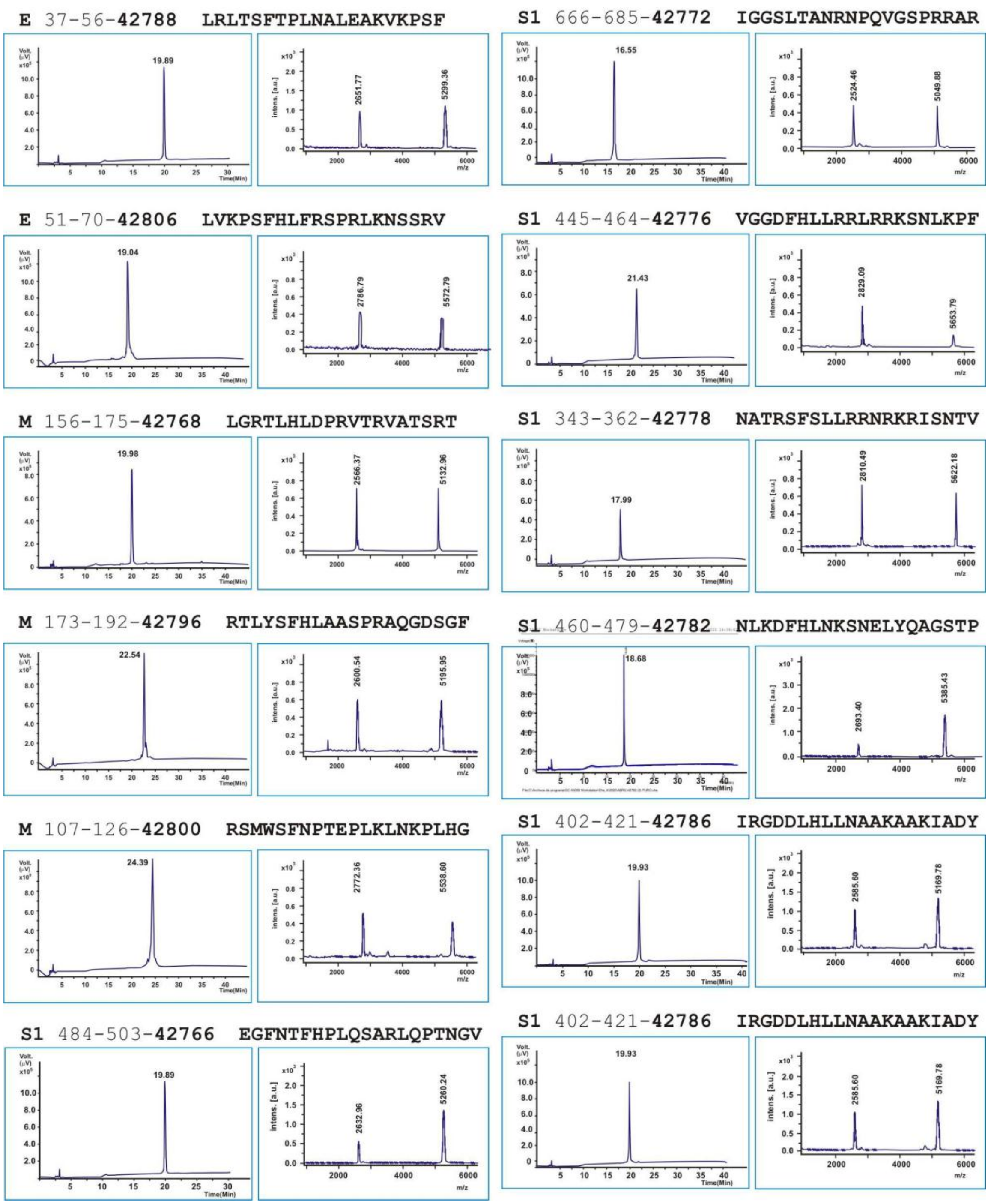

S1 107-126-**42802** **ITASFQVNTNPSLSASSVAS**

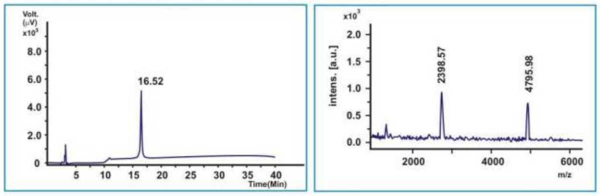

S1 691-710-**42804** **SIISYTMSKAGNVSKGVSNN**

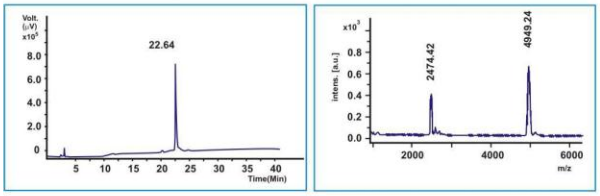

S1 61-80-**42810** **NVWTFHALHSAALGGTKRFD**

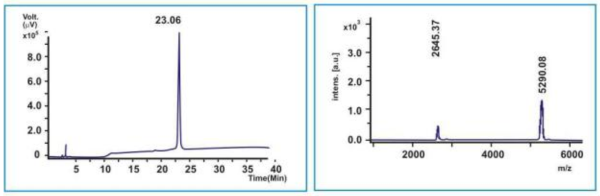

S1 105-124-**42812** **IFGTTLDALRAAKLKVNNAT**

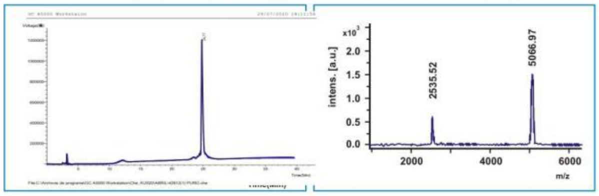

S1 129-148-**42814** **KVSEFQFTNPARLVRYHKN**

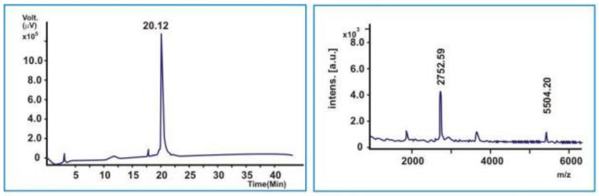

S1 152-171-**42816** **WMESEFHLYSSAPLGTREYV**

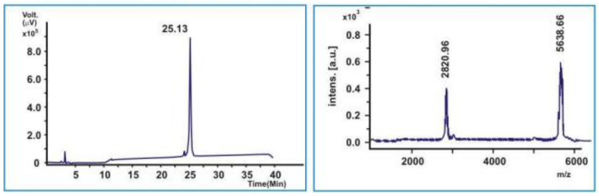

S1 170-189-**42818** **VSQPFHLDKAPRVGNFKNLR**

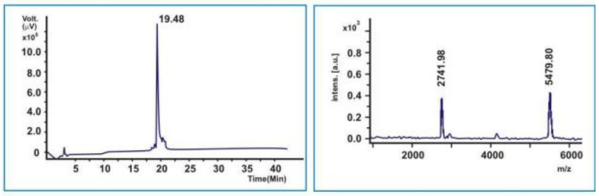

S1 190-219-**42820** **REFEFHLNNALRLKYSKHTP**

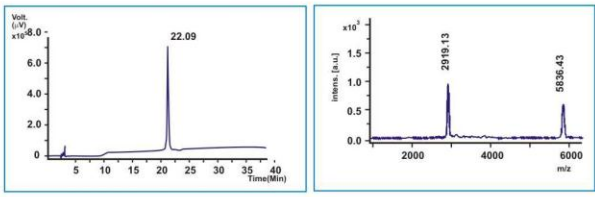

S1 220-239-**42822** **GFSAFLQLARALNLGKNITRF**

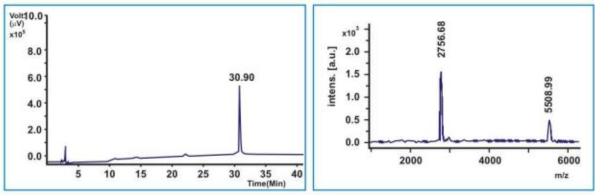

S1 235-254-**42824** **NIRTFQPLAKLRRSRLTPGD**

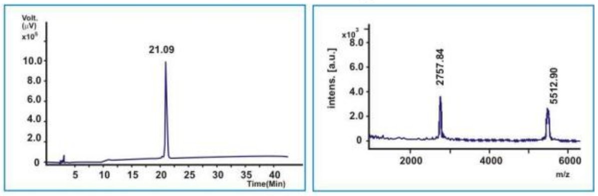

S1 386-405-**42826** **KLNDLTFDNKARSDRVIRGD**

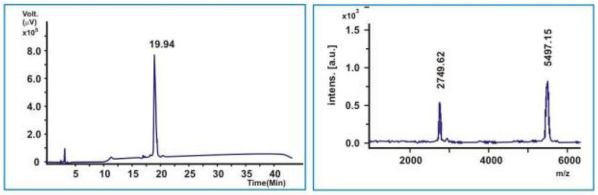

S1 439-458-**42828** **NNLADLNAGRANRLYRLFRK**

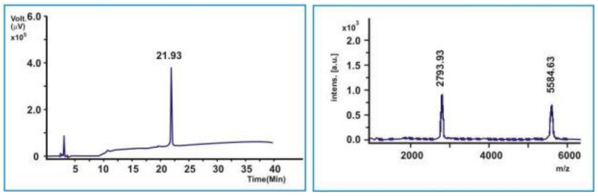

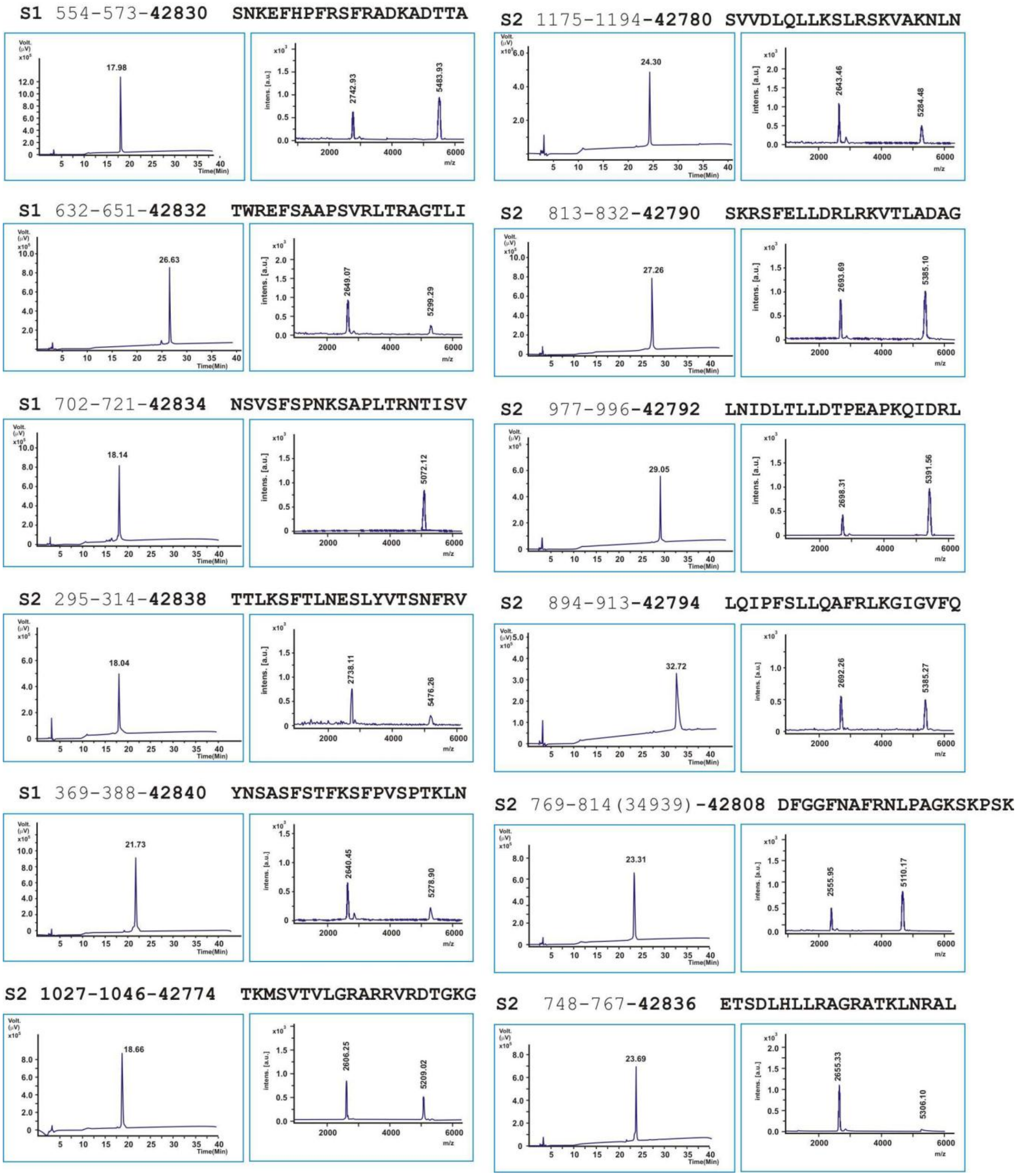

Supplementary Figure 1. Characterising synthetic SARS-CoV-2 peptides. HPLC and MS for all modified peptides, showing their location in the proteins, their amino acid sequences, elution times and MS molecular weights, highlighting >99% purity.

Supplementary Figure 2

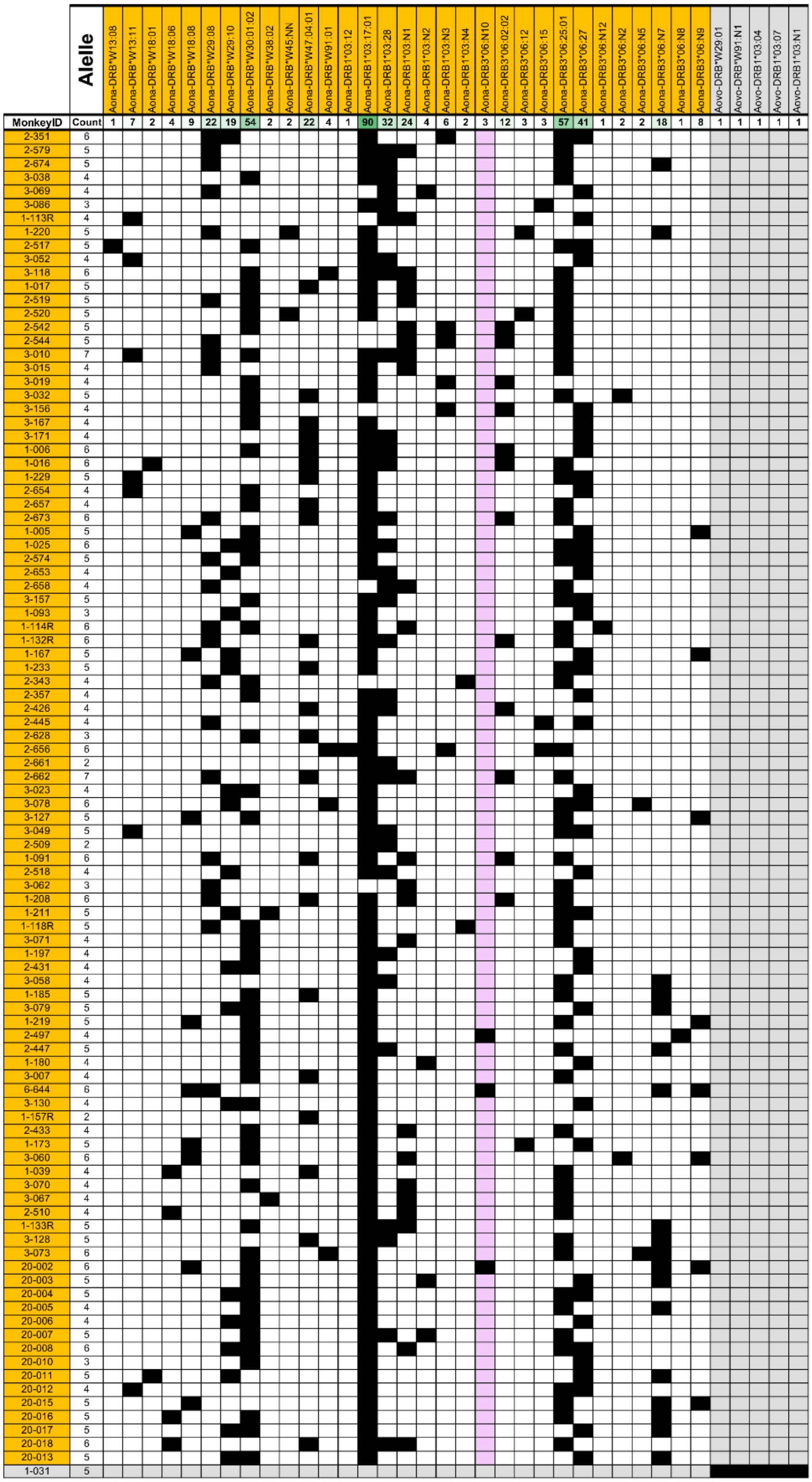

.

Supplementary Figure 2. MHC-DRB classification of the *Aotus* monkeys used in the trials

The Illumina NovaSeq sequencing system was used for typing the exon 2 amplicon. *A. nancymaae* monkey alleles are indicated in orange, *A. vociferans* in grey. The Figure indicates the number of monkeys in which the typed alleles were detected (rows) and the amount of alleles per monkey (columns). The green shading in the count column indicates the alleles most frequently occurring in the sample. The Aona-DRB3*06:N10 allele is highlighted in purple.

Supplementary Figure 3.

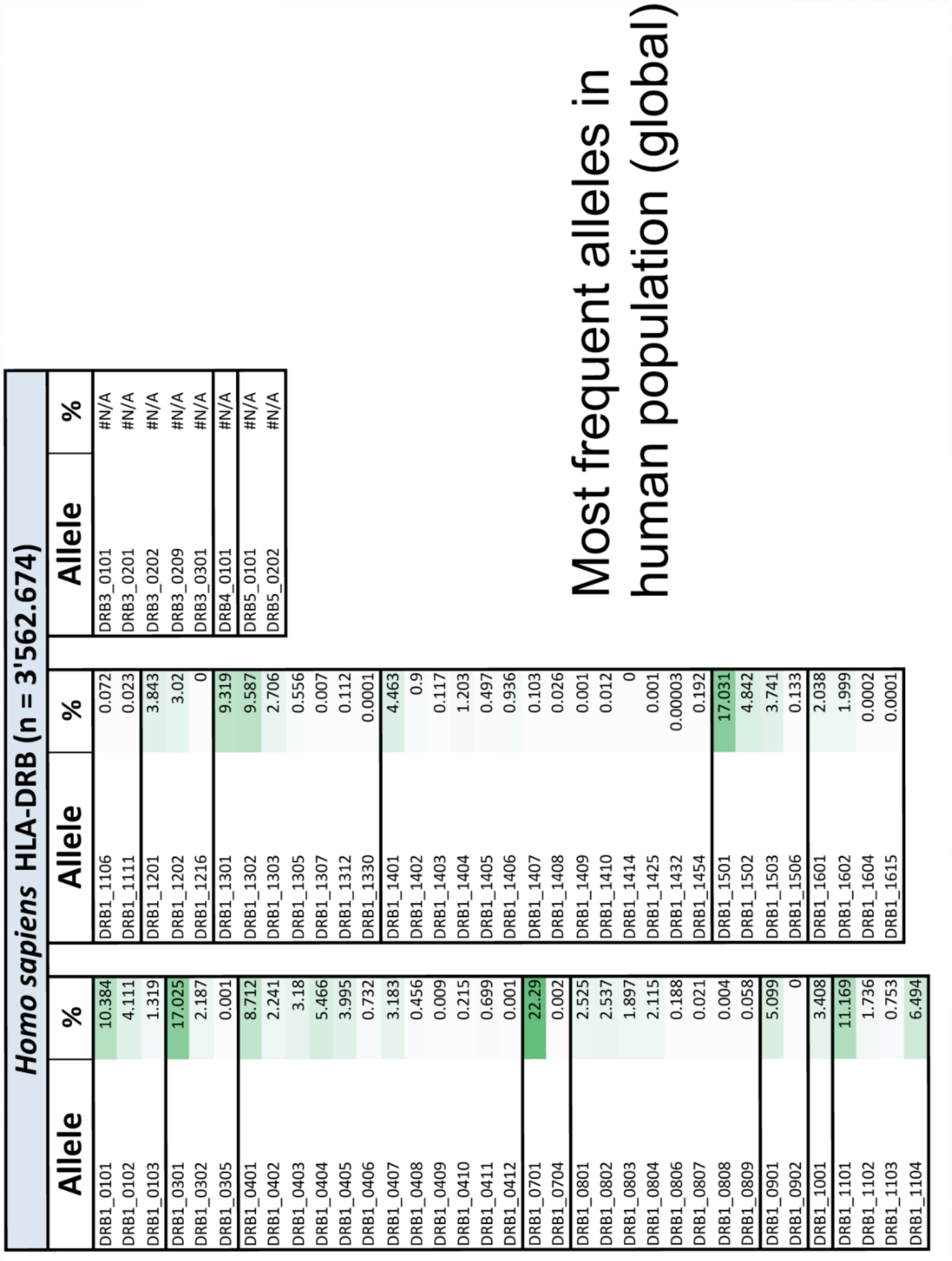

Homo sapiens HLA-DRB (n = 3'562.674)

Allele	%
DRB1_0101	10.384
DRB1_0102	4.111
DRB1_0103	1.319
DRB1_0301	17.025
DRB1_0302	2.187
DRB1_0305	0.001
DRB1_0401	8.712
DRB1_0402	2.241
DRB1_0403	3.18
DRB1_0404	5.466
DRB1_0405	3.995
DRB1_0406	0.732
DRB1_0407	3.183
DRB1_0408	0.456
DRB1_0409	0.009
DRB1_0410	0.215
DRB1_0411	0.699
DRB1_0412	0.001
DRB1_0701	22.29
DRB1_0704	0.002
DRB1_0801	2.525
DRB1_0802	2.537
DRB1_0803	1.897
DRB1_0804	2.115
DRB1_0806	0.188
DRB1_0807	0.021
DRB1_0808	0.004
DRB1_0809	0.058
DRB1_0901	5.099
DRB1_0902	0
DRB1_1001	3.408
DRB1_1101	11.169
DRB1_1102	1.736
DRB1_1103	0.753
DRB1_1104	6.494

Allele	%
DRB1_1106	0.072
DRB1_1111	0.023
DRB1_1201	3.843
DRB1_1202	3.02
DRB1_1216	0
DRB1_1301	9.319
DRB1_1302	9.587
DRB1_1303	2.706
DRB1_1305	0.556
DRB1_1307	0.007
DRB1_1312	0.112
DRB1_1330	0.0001
DRB1_1401	4.463
DRB1_1402	0.9
DRB1_1403	0.117
DRB1_1404	1.203
DRB1_1405	0.497
DRB1_1406	0.936
DRB1_1407	0.103
DRB1_1408	0.026
DRB1_1409	0.001
DRB1_1410	0.012
DRB1_1414	0
DRB1_1425	0.001
DRB1_1432	0.00003
DRB1_1454	0.192
DRB1_1501	17.031
DRB1_1502	4.842
DRB1_1503	3.741
DRB1_1506	0.133
DRB1_1601	2.038
DRB1_1602	1.999
DRB1_1604	0.0002
DRB1_1615	0.0001

Allele	%
DRB3_0101	#N/A
DRB3_0201	#N/A
DRB3_0202	#N/A
DRB3_0209	#N/A
DRB3_0301	#N/A
DRB4_0101	#N/A
DRB5_0101	#N/A
DRB5_0202	#N/A

Supplementary Figure 3. Frequently occurring alleles in the worldwide human population. HLA-DRB1* allele frequency information was determined by consulting the Allele Frequency Net database, having greater than 1% frequency in ethnic groups and geographical regions (69 alleles). Representative alleles were chosen for HLA-DRB alleles 3, 4 and 5 from the most frequent pocket profiles for each locus (8 alleles). Green shading indicates the most frequently occurring alleles in the worldwide human population.

Supplementary Figure 4.

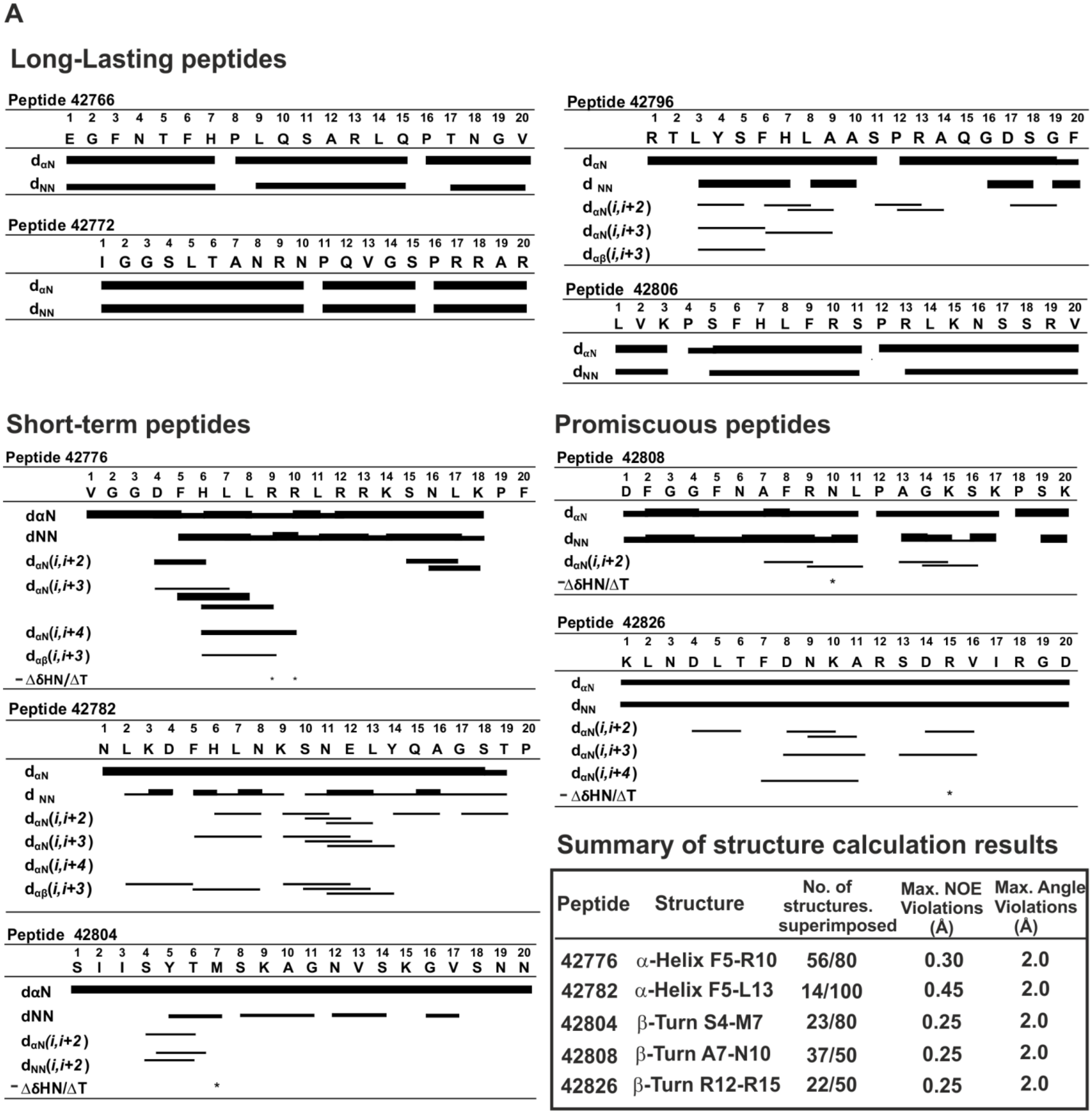

Peptide	Structure	No. of structures. superimposed	Max. NOE Violations (Å)	Max. Angle Violations (Å)
42776	α-Helix F5-R10	56/80	0.30	2.0
42782	α-Helix F5-L13	14/100	0.45	2.0
42804	β-Turn S4-M7	23/80	0.25	2.0
42808	β-Turn A7-N10	37/50	0.25	2.0
42826	β-Turn R12-R15	22/50	0.25	2.0

B

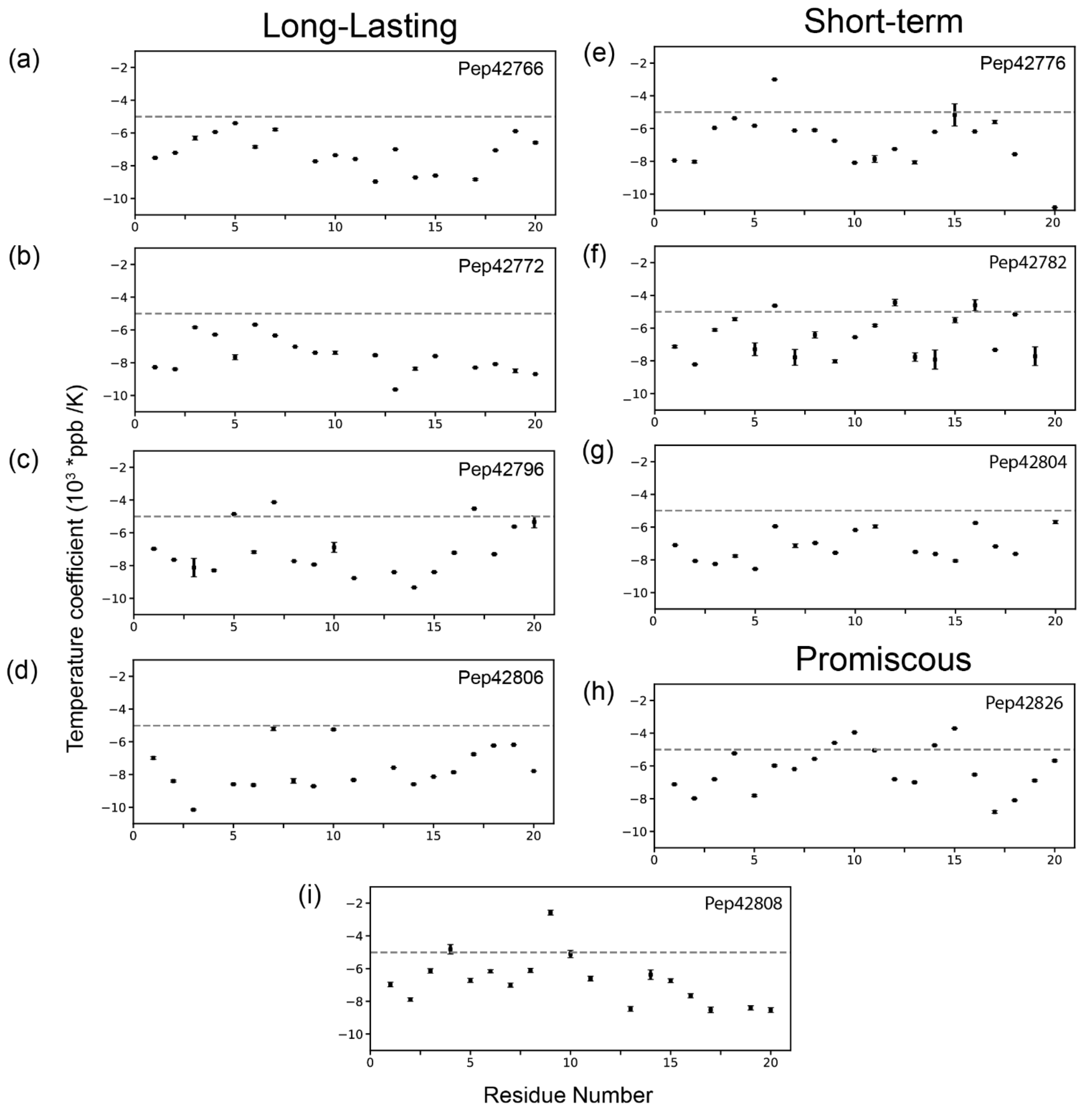
Long-Lasting
Short-term
Promiscous
(a)
Pep42766
(b)
Pep42772
(c)
Pep42796
(d)
Pep42806
(e)
Pep42776
(f)
Pep42782
(g)
Pep42804
(h)
Pep42826
(i)
Pep42808
Temperature coefficient (10³ *ppb /K)
Residue Number

C

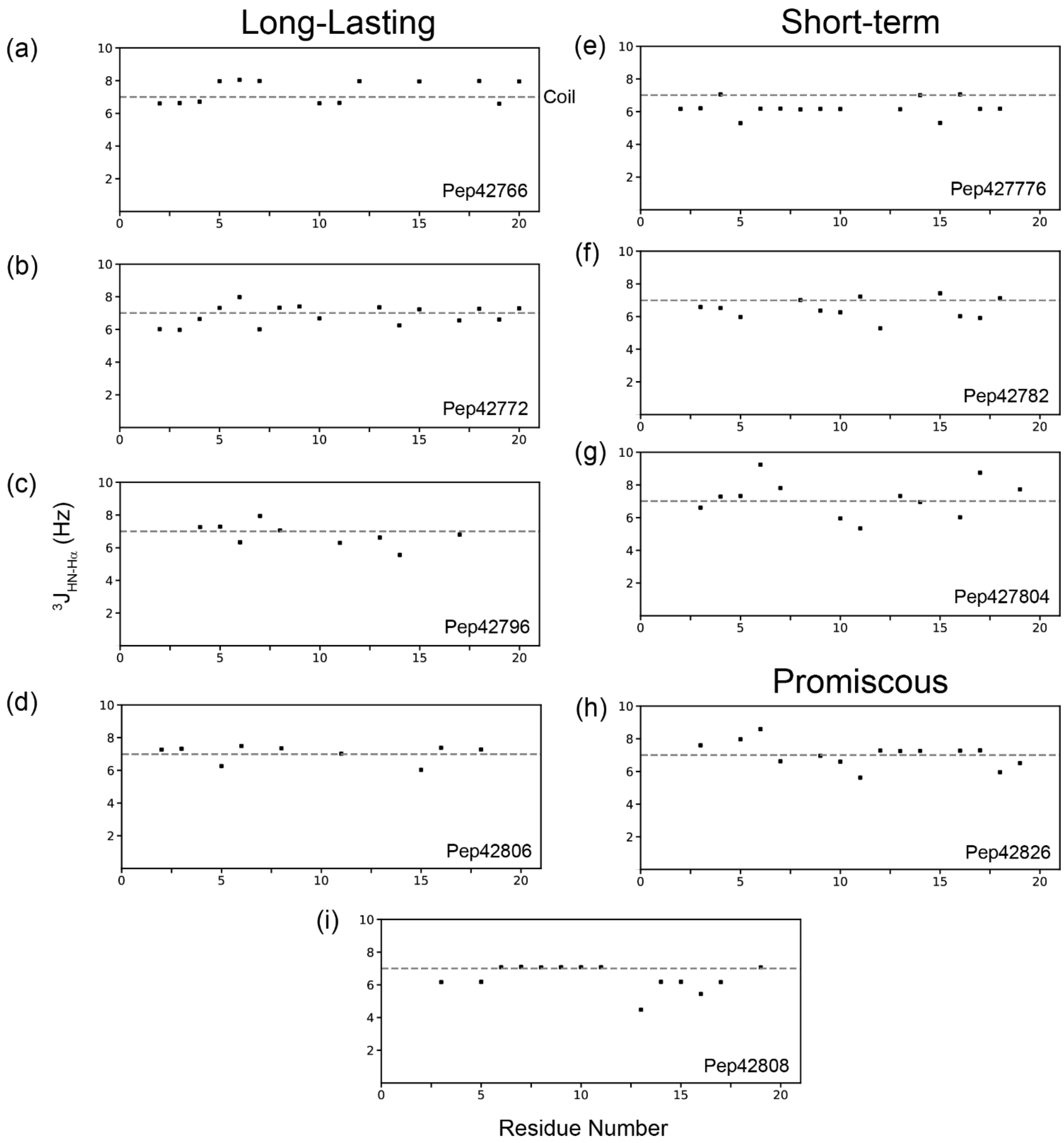

Long-Lasting
Short-term
Promiscous
(a)
(b)
(c)
(d)
(e)
(f)
(g)
(h)
(i)
Coil
Pep42766
Pep42772
Pep42796
Pep42806
Pep427776
Pep42782
Pep427804
Pep42826
Pep42808
$^3J_{HN\text{-}H\alpha}$ (Hz)
Residue Number

D

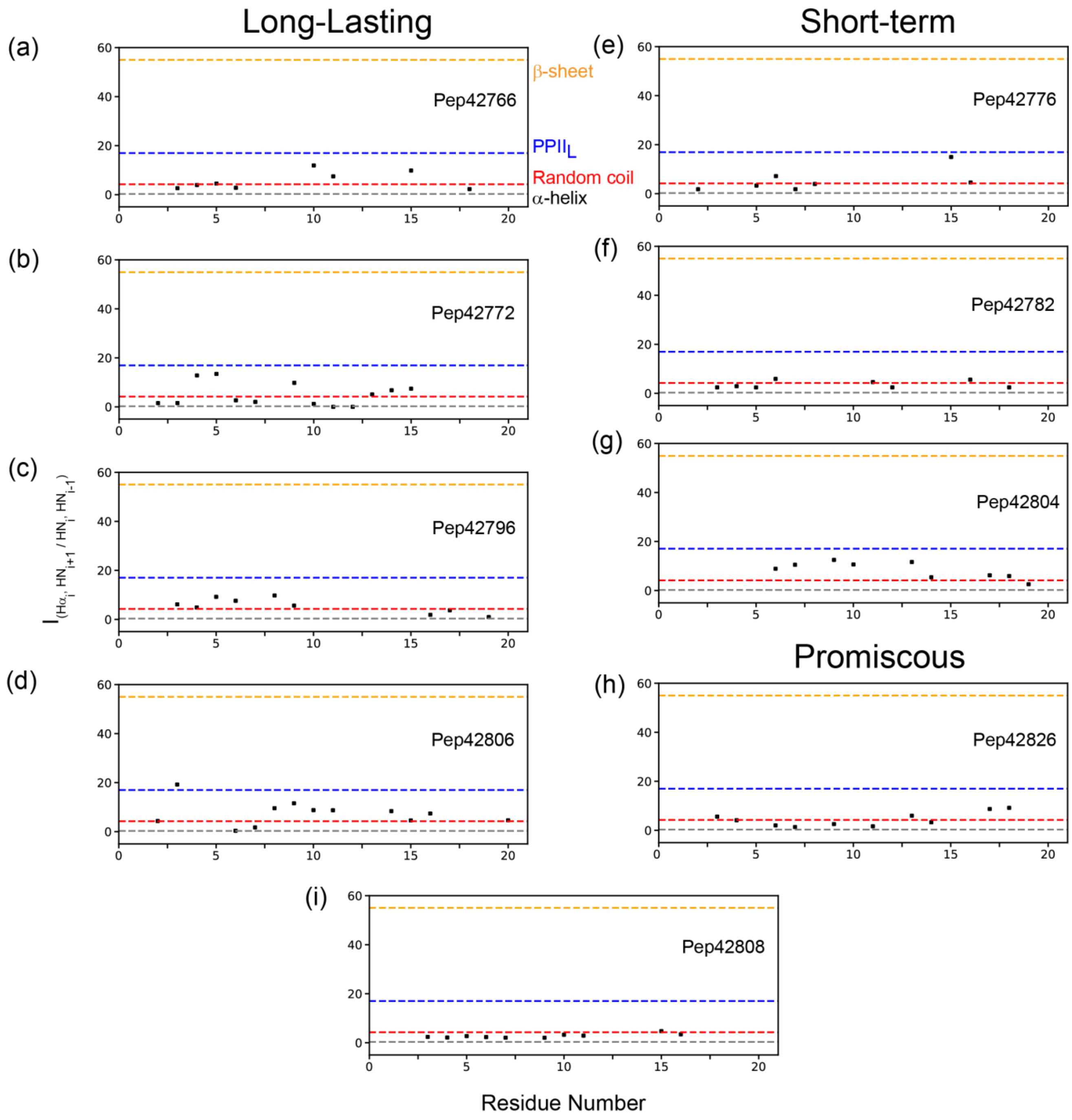
Long-Lasting
Short-term
Promiscous
(a)
Pep42766
β-sheet
PPII$_L$
Random coil
α-helix
(b)
Pep42772
(c)
Pep42796
(d)
Pep42806
(e)
Pep42776
(f)
Pep42782
(g)
Pep42804
(h)
Pep42826
(i)
Pep42808
$I_{(H\alpha_i, HN_i / HN_{i+1}, HN_{i-1})}$
Residue Number

Supplementary Figure 4. ^{1}H-NMR results for the peptides' 3D structure determination. (A) A summary of sequential medium range NOE connectivity (NOE intensities are represented by line thickness) for long-lasting, short-term and promiscuous peptides. NH protons having slow exchange rates (involved in H-bonds NH i ···Oi−4) are indicated by *. Results of the short-term and promiscuous peptides' structure calculations are shown in the table in the bottom right. (B) Temperature coefficient indicates intramolecular H-bonding propensity in long-lasting, short-term and promiscuous peptides. Values greater than -5 ppb/K (dotted line) are residues involved in potential H-bond formation. (C) ^{3}J HN-Hα coupling constants regarding each peptide's primary sequence. A value close to 7 Hz (represented as -- line) indicates random coil propensity. (D) HN-Hα intensity ratio indicates structural formation probability values. The yellow dotted line represents β-sheet, blue $PPII_L$, red random coil and grey α-helix tendency. (a), (b), (c), (d), (e), (f), (g), (h) and (i) correspond to **42766**, **42772**, **42796**, **42806**, **42776**, **42782**, **427804**, **42826** and **42808** peptides respectively in NMR studies results.
